# An inducible gene from glycoside hydrolase one family of *Plutella xylostella* decreases larval survival when feeding on host plant

**DOI:** 10.3389/fphys.2022.1013092

**Published:** 2022-10-20

**Authors:** Wei Chen, Yuhong Dong, Ling Zheng, Yingfang Lai, Feifei Li, Li Zhou, Beibei Wang, Minsheng You, Weiyi He

**Affiliations:** ^1^ State Key Laboratory for Ecological Pest Control of Fujian and Taiwan Crops, Institute of Applied Ecology, Fujian Agriculture and Forestry University, Fuzhou, China; ^2^ Ganzhou Key Laboratory of Greenhouse Vegetable/College of Life Sciences, Gannan Normal University, Ganzhou, China; ^3^ International Joint Research Laboratory of Ecological Pest Control, Ministry of Education, Fujian Agriculture and Forestry University, Fuzhou, China; ^4^ Ministerial and Provincial Joint Innovation Centre for Safety Production of Cross-Strait Crops, Fujian Agriculture and Forestry University, Fuzhou, China

**Keywords:** *Plutella xylostella*, insect myrosinase, CRISPR/Cas9, plant defense, toxifying gene

## Abstract

Glycoside hydrolase family 1 (GH1) members exhibit a broad substrate spectrum and play important roles in insect-plant interactions, such as the defensive β-glucosidase and β-thioglucosidase (so-called myrosinase). However, knowledge about the expression profiling and function of glycoside hydrolase family 1 members in a specialist pest of crucifers *Plutella xylostella* is still limited. In this study, 13 putative glycoside hydrolase family 1 members of *P. xylostella* were identified based on the sequence characteristics, while no myrosinase activity was detectable in *P. xylostella* using gas chromatography-mass spectrometry (GC-MS). Expression profiling of these glycoside hydrolase family 1 members identified the midgut-specific gene *Px008848* that is induced by host plant. Further experiments revealed that the *in vitro* expressed Px008848 protein had β-glucosidase activity and the survival rate of the larvae feeding on wounded *Arabidopsis thaliana* leaves declined when leaves were treated with purified Px008848 protein. When CRISPR/Cas9-based homozygous mutant larvae of *Px008848* and wild-type larvae were respectively transferred onto the *A. thaliana*, the larval survival rate of the mutant larvae was significantly higher than that of the wild-type individuals. Our work showed that certain insect glycoside hydrolase family 1 gene may have negative effect on the development of larvae feeding on the host plant, which broadened our understandings on the evolutionary function of this gene family in the insect-plant interaction.

## Introduction

Glycoside hydrolases (GHs), also known as glycosidases, are a class of enzymes that catalyze the hydrolysis of O-, N- and S-linked glycosides to form sugar hemiacetal (or hemiketal) and the corresponding free aglycon (Rye & Withers, 2000). To date, 170 documented glycoside hydrolase families (GH1-GH170) have been identified in living organisms, in which they play a variety of functions in the hydrolysis and synthesis of sugars and glycol conjugates (Cantarel et al., 2009). Among them, GH1 members, such as defensive β-glucosidase and β-thioglucosidase (so-called myrosinase), play important roles in insect-plant interactions, which have led to coevolution between insects and plants (Morant et al., 2008; Ketudat-Cairns & Esen, 2010; Pentzold et al., 2014).

GH1-centered defense systems are effective and safe strategies commonly adopted in the plant kingdom: glucoside substrates and corresponding GH1 members are stored in distinct subcellular compartments of plants (Koroleva et al., 2000; Zagrobelny et al., 2004). The well-characterized glucoside components in various plants include alkaloid glucosides, benzoxazinoid glucosides, cyanogenic glucosides, iridoid glucosides and salicinoids, etc (Desroches et al., 1995; Møller 2010; Dobler et al., 2011; Dixon et al., 2012; Pentzold et al., 2014). When the plants are under attack by insect herbivores, these glucoside components come into contact with endogenous β-glucosidases of GH1 family, and subsequently produce toxic aglycones to deter the pests. More intriguingly, although the digestive enzymes from insects also contain β-glucosidases which react with these glucoside substrates after ingestion of plant material, some insects circumvent formation of toxic aglycones by virtue of lowering their endogenous β-glucosidases activities (Desroches et al., 1997; Ballhorn et al., 2010; Pankoke et al., 2012). Even so, little was known about the role of endogenous GH1 β-glucosidase in specialist insects under an insect-plant interaction context.

As another member of GH1 family, the myrosinases are the important component for the activation of “mustard oil bombs”, which is a chemical defense utilized by cruciferous plants to resist the insect herbivore attack (Kissen et al., 2009). The substrate glucosinolates (GSs), also known as mustard oil glucosides, are nitrogen- and sulfur-containing plant secondary metabolites that are enriched in the order Brassicales (Fahey et al., 2001; Halkier & Gershenzon, 2006; Morant et al., 2008; Bones & Rossiter, 2010). Under normal circumstances, GSs and myrosinases are localized in different cell compartments in cruciferous plants (Kissen et al., 2009). When crucifers are attacked by insect herbivores, the plant cells are damaged (Kliebenstein et al., 2005) and a variety of toxic hydrolytic products of the GSs are generated under the catalysis of myrosinases, including isothiocyanates (ITCs), nitriles, epithionitriles and thiocyanates (Textor & Gershenzon, 2009). Similarly, some specialist insects also “copy” the plant’s “mustard oil bombs” defensive system through separated storage of plant-derived GSs and evolution of insect-specific myrosinase enzymes. It has been reported that insect herbivores of different orders can not only sequester various GSs of host plants (Müller et al., 2002; Müller & Wittstock, 2005), but also activate the GSs by insect-specific myrosinase to defend themselves against natural enemies (Müller, 2009; Opitz & Müller, 2009; Erb & Robert, 2016; Robert et al., 2017; Yang et al., 2021), such as cabbage aphid *Brevicoryne brassicae* (Bridges et al., 2002; Jones et al., 2002; Kazana et al., 2007) and flea beetle *Phyllotreta striolata* (Beran et al., 2014). Despite this, some insect GH1 genes of unknown function were confusedly annotated as myrosinase-like genes. It is probably because myrosinases and β-glucosidases shared amino acids known to be involved in substrate binding and hydrolysis and presented mixed evolutionary patterns (Jones et al., 2002; Husebye et al., 2005; Huber et al., 2021). Therefore, it is fundamental to carry out experimental verification to clarify the function of insect GH1 genes in insect-host plant coevolution.

Known as one of the world’s major insect pests (Zalucki et al., 2012), the cruciferous specialist diamondback moth (*Plutella xylostella*) contained a total of 13 GH1 family members that have been annotated as myrosinase-like genes (You et al., 2013). Considering that *P. xylostella* uses glucosinolate sulfatases (GSSs), which can hydrolyze all major classes of glucosinolates to form desulfo-glucosinolates, to compete with myrosinase of the host plants (Ratzka et al., 2002; You et al., 2013; Ma et al., 2018; Heidel-Fischer et al., 2019; Chen et al., 2022), therefore, it would be interesting to explore whether these 13 GH1 genes in *P. xylostella* encode functional myrosinases. If not, the functional roles of these GH1 genes as possible β-glucosidases would be further explored in insect-plant interaction.

In this study, endogenous myrosinase activities in two *P. xylostella* strains, FZ and AD reared on radish seedlings or artificial diet, respectively, were firstly excluded based on gas chromatography-mass spectrometry (GC-MS) assays. Expression profiling of the 13 putative GH1 genes identified the larva-specific and midgut-specific candidates. Among them, one candidate *Px008848* which could be induced by host plants, was further subjected to functional characterization for myrosinase and β-glucosidase activity using a heterologous protein expression system and its potential roles in the host adaptability of *P. xylostella* using CRISPR/Cas9 technology. Our work reveals the absence of functional myrosinase in *P. xylostella* and provides important clues for understanding the functional roles of insect GH1 members in the context of insect-plant interactions.

## Materials and methods

### Insect and plant rearing

Under an insect rearing cage (36 × 36 × 50 cm), two insecticide-susceptible *P. xylostella* strains, named the FZ and AD strains, were reared on four-day-old radish seedlings of *Raphanus sativus* var. Nanpanzhou (contact with plant myrosinase) and artificial diet (avoidance of plant myrosinase), respectively (You et al., 2013; Huang et al., 2017). The compound of artificial diet consists of 8% yeast extract, 2.4% agar, 15% wheat germ powder, 0.4% complexed vitamins (powder mixture of vitamins A, B_1_, B_2_, B_6_, B_12_, C, D_3_ and E), 0.4% sorbic acid, 0.4% paraben and 4% sucrose. All ingredients were mixed with 250 ml water and boiled in a microwave oven, followed by supplement of two drops of linoleic acid. Environmental conditions for insect rearing included temperature at 25 ± 1°C, light:dark cycle L:D = 16:8 h and relative humidity (RH) = 65 ± 5%. All adults of *P. xylostella* were fed 10% honey water. The radish seedlings and *A. thaliana* Col-0 ecotype plants used for the feeding assays were cultivated in mixed soil (vermiculite: humus = 1:1) for 4 days or 6 weeks, respectively. The cultivation parameters were consistent with the conditions for insect rearing.

### Coding sequence cloning

Total RNAs were extracted from samples of the FZ and AD larvae using TRIzol^®^ reagent (Invitrogen, Guangzhou). The corresponding cDNAs were synthesized using Hifair®II 1st Strand cDNA Synthesis Kit (YEASEN, 11121ES60, China). The Phanta Max Super-Fidelity DNA Polymerase (Vazyme, #P505, China) was used for PCR amplification and PCR products of were directly sequenced by Sanger sequencing. Primer pairs were designed based on the sequence information provided in genome database of *P. xylostella* (DBM-DB) (Tang et al., 2014), which was shown in Supplementary Table S1. Four full-length coding sequences (CDSs, *Px006941*, *Px008848*, *Px008849* and *Px006054*) and three partial sequences (*Px009427*, *Px009428* and *Px000291*) of the putative GH1 genes were obtained.

### Phylogenetic analysis

The deduced protein sequences of insect β-glucosidases and insect/plant myrosinases, which have been functionally verified, and 13 putative GH1 genes of *P. xylostella* were used for the alignment. All CDSs sequences were manually corrected based on available transcriptome data of *P. xylostella* on Lepbase (http://blast.lepbase.org/). The sequences of four full-length CDSs of putative GH1 genes were further corrected by PCR. Besides, two β-glucosidases from *Melolontha* (Huber et al., 2021) and *Manduca sexta* (FK816842), four insect myrosinases from *B. brassicae* (Q95X01), *Acyrthosiphon pisum* (XP_001946492 and XP_001952406) and *P. striolata* (KF377833), and two plant myrosinases from *A. thaliana* (NP_851077 and NP_568479), the functions of which have all been verified, were also used for the alignment. The sequences were aligned using ClustalW and then manually adjusted to equal length. Phylogenetic analysis was performed using Maximum Likelihood method (LG + G + I model) based on an optimal model analysis searching embedded in MEGA (version 7.0.1), and the bootstrap with 1,000 replicates was presented. The information of amino acids known to be involved in substrate binding and hydrolysis of myrosinases and β-glucosidases was sourced from previous reports (Davies and Henrissat, 1995; Beran et al., 2014; Huber et al., 2021).

### Measurement of qualitative allyl-isothiocyanate using chromatography-mass spectrometry

Samples from different developmental stages and tissues of the fourth instar larvae of the FZ strain and gut contents from the fourth instar larvae of the AD strain were used to extract total protein following the instructions of the animal tissue total protein (non-denatured) extraction kit (Invent Biotechnologies, United States). A kit based on the BCA method (YEASEN, 20201ES76, China) was used to quantify the protein concentration. The production of allyl-isothiocyanate (AITC) was collected using solid-phase micro-extraction fiber sampler (Supelco, 57330-U, United States) and examined by GC-MS (Agilent, 7890B-Agilent, 5977A, United States). The standard for AITC was commercially available (Sigma-Aldrich, W203408), and its retention time shown in GC-MS was used as the reference. The production of AITC from the reaction of plant myrosinase (Sigma-Aldrich, T4528) (20 µg) and sinigrin (10 mM) was set as a positive control. Details of the operations, volatile collection and program in the GC-MS experiment have been described in our previous work (Chen et al., 2020).

### Sample collection for cDNA preparation and gene expression profiling

To investigate the expression profiling of 13 GH1 genes in *P. xylostella*, different developmental stages and larval tissues of the FZ strain were collected. The developmental stages included eggs (approximately 200 per replicate), the first to fourth instar larvae and mature pupae and adults of both sexes (six individuals per replicate). Larval tissues, including the salivary gland, silk gland, Malpighian tubule, fat body and midgut, were dissected from the 30 individuals of the fourth instar larvae for each biological replicate.

To identify the candidates of GH1 genes that could respond to host transfer, the AD larvae were subjected to hour-course (short-term) and generation-course (long-term) challenges from an artificial diet to a host plant. For short-term challenges, the newly emerged fourth instar AD larvae were separated into two groups, one reared on the original artificial diet and the other transferred onto radish seedlings. Midgut samples without the gut content were dissected from the corresponding larvae at 8 h, 24 h and 48 h after treatment. For long-term challenges, the newly emerged first instar AD larvae were transferred onto the host plant for two consecutive generations, named Trans-G1 and Trans-G2. AD and FZ larvae reared on the original diet were used as controls. The midgut samples of the six fourth instar larvae of Trans-G1, Trans-G2, AD and FZ were dissected for subsequent experiments. To further verify the gene expression of *Px008848* was also induced by *A. thaliana*, the midgut samples without the gut content were collected from the corresponding larvae at 48 h after feeding on *A. thaliana* or artificial diet.

For all samples mentioned above, three biological replicates were collected, and total RNA was isolated using TRIzol^®^ reagent (Invitrogen, Guangzhou) according to the manufacturer’s instructions. The Hifair®II 1st Strand cDNA Synthesis Kit (YEASEN, 11121ES60, China) was used for the synthesis of cDNA from the collected samples, individually. Quantitative real-time PCR (qRT-PCR) was conducted using Go Taq^®^ qPCR Master Mix (Promega, A600A, United States) in a CFX96 Touch Real-Time PCR Detection System (Bio-Rad, United States) with a program of 95°C for 3 min and 40 cycles at 9°C for 15 s, 55°C for 15 s and 72°C for 30 s. Primer information for the qRT-PCR is shown in Supplementary Table S1. The ribosomal protein gene *L32* was used as the internal reference gene (Chen et al., 2019). All the products of qRT-PCR with a length of approximately 150 bp were verified by Sanger sequencing.

### Western blotting

Samples from different larval stages and different tissues of the fourth instar larvae of AD were used. In addition, midgut samples from larvae under short- or long-term plant challenges, including AD-24 h, AD-48 h and Trans-G1, were collected for protein extraction. Total proteins of the samples from three biological repeats were extracted by an animal tissue total protein (denaturation) extraction kit (Invent Biotechnologies, Minute, United States) according to the manufacturer’s instructions. Sodium dodecyl sulfate-polyacrylamide gel electrophoresis (SDS-PAGE), semidry transfer of the protein and Western blotting were performed according to Chen et al. (2020). The polyclonal antibody of Px008848 was produced by ABclonal Biotechnology Company (Wuhan, China). An amount of 20 µg protein was loaded for each sample.

### Prokaryotic expression, purification and renaturation of *Px008848*

Primers with *BamH*I and *Xho*I enzyme recognition sites were used for amplification of the *Px008848* CDS (Supplementary Table S1). The CDS was subcloned into the expression vector pET32a (laboratory preservation) to form a recombinant plasmid, which was transformed into *Escherichia coli* BL21 (DE3) competent cells (TransGen, Beijing, China) for subsequent protein expression. To optimize the protein expression, the concentrations of the inductive agent isopropyl β-D-thiogalactoside (IPTG) and the temperature were adjusted. The final condition for protein expression was 0.5 mM β-D-thiogalactoside at 20°C for 16 h. Harvested cells were disrupted in lysis buffer (50 mM PBS, pH 7.4 and 0.5 M NaCl) under sonication on ice (300 W, interval of on-off for 10 s, 30 times). The supernatant and pellets were separately collected by centrifugation (12,000 × *g* for 20 min at 4°C). Considering that the expressed protein was largely presented in pellets, the pellets were collected and suspended in 8 M urea. The collections were loaded on a pre-equilibrated Ni-NTA purification column (GenScript, L00250, China), and the recombinant proteins were eluted by NTAU buffer (50 mM PBS, pH 7.4, 0.5 M NaCl, 8 M urea, and X mM imidazole, where X represents 5, 10, 20, 30, 40, 50 or 200). The elution was dialyzed twice in buffer containing 20 mM Tris-HCl (pH 8.0), 10 mM MgCl_2_, 1 mM DTT and multistep renaturations were performed in the same buffer with a decreasing gradient of urea (8, 6, 4, 2 and 0 M). The collections were verified by 12% SDS-PAGE electrophoresis and quantified using bovine serum albumin (BSA) standard protein.

### Sequence analysis and enzyme activity detection of Px008848

The *N*-glycosylation and GalNAc-type *O*-glycosylation sites in deduced protein sequences of Px008848 were predicted by using online software NetNGlyc (https://services.healthtech.dtu.dk/service.php?NetNGlyc-1.0) and NetOGlyc (https://services.healthtech.dtu.dk/service.php?NetOGlyc-4.0), receptivity. An equal amount (20 µg) of the protein from each sample or the heterogeneously expressed Px008848 protein was added into the reaction mixture containing glucosinolate sinigrin (10 mM, Sigma-Aldrich, 85,440), which were then incubated for 15 min at room temperature in a sealed vial. The detection of myrosinase activities was performed by using the same method as described above. For detection of β-glucosidase activity, 10 µg of Px008848 protein was incubated with p-nitrophenyl β-d-glucopyranoside for 30 min at 37°C. The product detection of p-nitrophenyl and activity calculation was performed for four replicates according to the manufacturer’s instructions provided in the kit (Solarbio, BC2560). One unit (U) of enzyme activity was defined as 1 nmol p-nitrophenol produced per mg of tissue protein per hour.

### CRISPR/Cas9-based homozygous mutant strain of Px008848

Based on searching the N_20_NGG sequence for screening appropriate sgRNA candidates, two sgRNAs located in the third exon of *Px008848* with an interval of 124 bp were designed and synthesized (Supplementary Table S1). The procedures for the preparation of sgRNAs and embryo microinjection were performed as previously described (Chen et al., 2020). Mixtures of the two sgRNAs and Cas9 protein were co-injected to generate a mutation in *Px008848*. Approximately 200 newly laid eggs of the wild-type AD strain were used for injection, and each adult (G0) developed from the injected eggs was crossed with a wild-type adult of the opposite sex to produce progeny. Each G0 adult was used for DNA extraction and PCR to test the mutation of the gene region targeted by sgRNAs using the primer pairs provided in Supplementary Table S1. The progeny (G1) derived from each single-pair mating of G0 adults were maintained when their female or male parent was identified as a mutant based on Sanger sequencing. For the sibling adults of each G1 population, self-crossing was carried out based on random single-pair mating, which was repeated for successive generations until homozygous mutant alleles appeared in the paired adults. After that, individuals of the progeny were randomly tested using PCR to confirm the establishment of the homozygous *Px008848* mutant strain.

### Bioassays

To explore the effect of purified Px008848 on larval survival rate of *P. xylostella*, each of ten leaves on *A. thaliana* plant was punched once using sterilized pipette tip, and the wounds were treated with 10 µL of 1 × PBS or 2 mg/ml Px008848 protein for 15 min. A total of 60 individuals of the third instar larvae were then transferred onto daily changed *A. thaliana* plants under each treatment, and the daily larval survival rate was recorded. To further identify the function of Px008848, the wild-type AD strain and the mutant strain of *Px008848* were reared on the *A. thaliana* Col-0 ecotype. Each treatment consisted of three biological replicates, with each replicate containing 20 individuals of the third instar larvae. Biological parameters of the larval survival rate and pupal weight were recorded and analyzed.

### Statistical analysis

The 2^−∆Ct^ method was employed to quantify the gene expression level (Livak & Schmittgen, 2002). The relative gene expression values and biological parameters were statistically analyzed by SPSS software (version 21.0). One-way ANOVA followed by Tukey’s HSD post hoc test and student *t* test were used for the analysis of the data (*p* < 0.05). The log-rank test was used to analyze the percent survival on *A. thaliana* plant treated with PBS or Px008848 protein. The figures were drawn by GraphPad Prism (version 5.0.1).

## Results

### Sequence analyses of 13 glycoside hydrolase family 1 genes and exclusion of myrosinase activities

**FIGURE 1:**
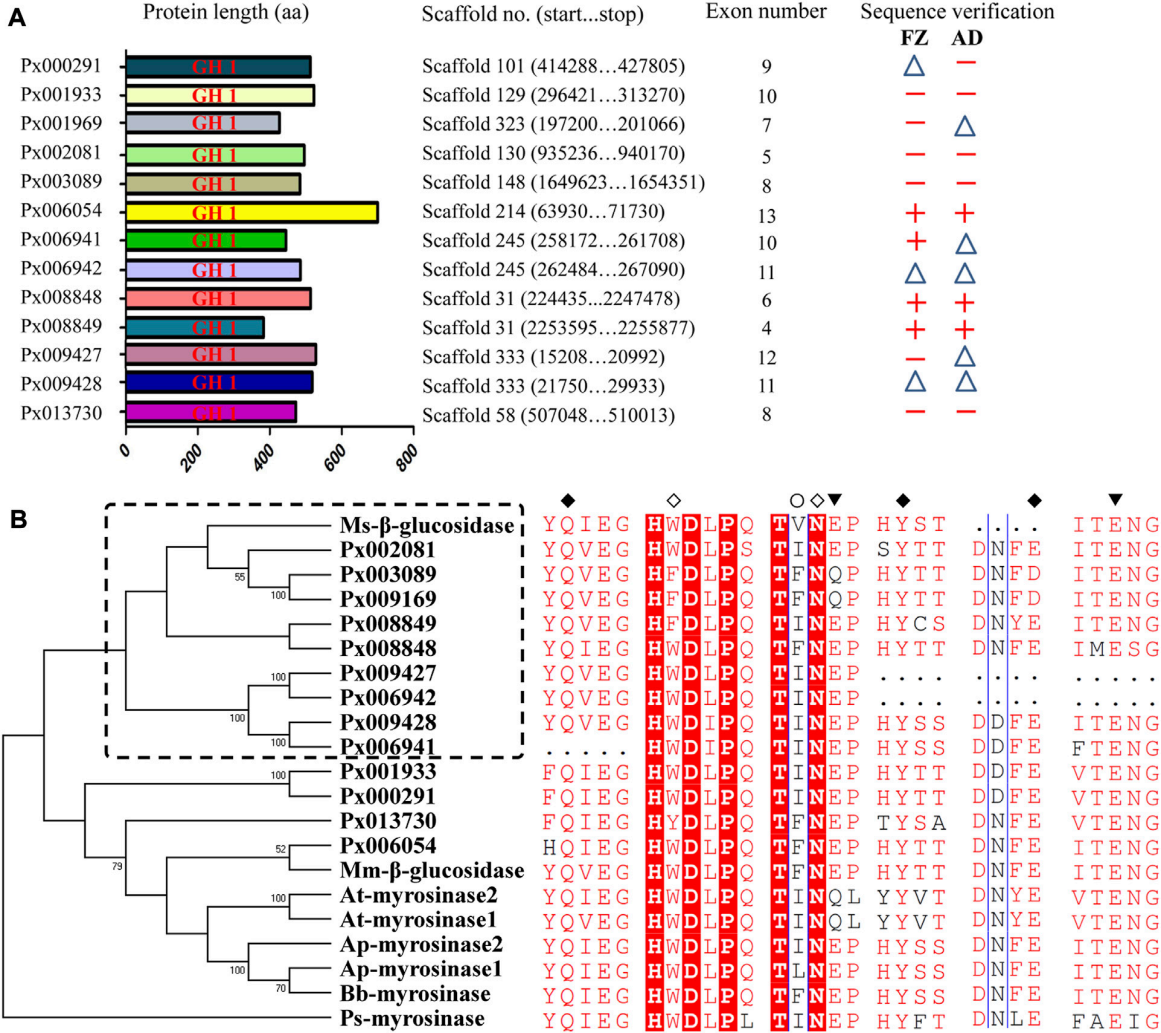
Characteristics and phylogenetic analysis of 13 putative GH1 genes in *P. xylostella*. **(A)** Deduced proteins of the 13 putative GH1 genes in *P. xylostella*. The sequences were drawn to scale with genomic information indicated. The corresponding coding sequences (CDSs) of the two strains were validated by PCR. +: full-length amplification of CDS; △: partial-length amplification of CDS (>300 bp); −: failed amplification. **(B)** Phylogenetic analysis based on deduced protein sequences of the 13 GH1 genes and known β-glucosidases and myrosinases. Ms-β-glucosidase: β-glucosidase of *M. sexta* (FK816842); Mm-β-glucosidase: β-glucosidase of *M. melolontha* (Huber et al., 2021); Bb-myrosinase: β-glucosidase of *B. brassicae* (Q95X01); Ap-myrosinase1 and Ap-myrosinase2: myrosinases of *A. pisum* (XP_001946492 and XP_001952406); At-myrosinase1 and At-myrosinase2: myrosinases of *A. thaliana* (NP_851,077 and NP_568,479); Ps-myrosinase: myrosinase of *P. striolata* (KF377833). ◇: Amino acids known to be involved in substrate binding of myrosinases (W and N); ○: Amino acids known to be involved in substrate recognition of β-glucosidases (I); ◆: Amino acids known to be involved in substrate binding shared by myrosinases and β-glucosidases (Q, Y and E); ▼: Two glutamic acids known to be involved in substrate hydrolysis shared by myrosinases and β-glucosidases (the left and middle E denoted an acid and nucleophile/base catalyst, respectively).

Through searching the genome database of *P. xylostella* (FZ strain), a total of 13 GH1 genes, originally annotated as myrosinase-like, were identified. To further confirm the correctness of 13 putative GH1 sequences, the corresponding CDSs were manually corrected by searching the available transcriptome data of *P. xylostella* on Lepbase (http://blast.lepbase.org/). Full or partial CDSs of the 13 GH1 genes were also validated using PCR, and polymorphisms were observed in the CDSs between the FZ and AD strains (Supplementary Figure S1 and Supplementary Table S2). The deduced protein sequences of 13 putative GH1 genes are approximately 500 aa in length and each of them contained one GH1 domain (Figure 1A). Phylogenetic analysis based on the deduced protein sequences of 13 GH1 genes and the known myrosinases and functional β-glucosidases in other species showed a mixed evolutionary pattern between the two classes of enzymes (Figure 1B). The identified insect GH1 genes seem to be evolved independently and diverge from plant myrosinases. Although the 13 putative GH1 showed credible relationship with the selected myrosinases and β-glucosidases, most of the putative GH1 genes (9/13) were clustered with the β-glucosidase of *M. sexta*. We found that some amino acids known to be involved in substrate binding and hydrolysis are conserved between insect myrosinases and β-glucosidases. The amino acids glutamine and tyrosine (Q, Y and E) known to be involved in substrate binding shared by myrosinases and β-glucosidases are identical among the ten putative GH1 genes, apart from three incomplete GH1 genes (*Px006941*, *Px006942* and *Px009427*) (Figure 1B).

To measure myrosinase activity on the basis that myrosinase can hydrolyze sinigrin to produce volatile AITC, GC-MS method was used to detect the myrosinase activity in *P. xylostella* larvae, pupae and adults of the FZ strain reared on plant. The GC-MS results showed that myrosinase activity could only be detected in the larvae stage (Supplementary Figure S2). Subsequently, we tested the distribution of myrosinase activities in different tissues/parts of the FZ larvae and the myrosinase activity was only detected in the gut content of FZ larvae (Supplementary Figure S3). To confirm that the myrosinase activity was from plant but not insect itself, gut contents from AD larvae reared on artificial diet were tested as well. However, no activity was detected in the gut contents of AD strain, which did not contain any plant tissues (Supplementary Figure S3).

### Expression profiling of putative glycoside hydrolase family 1 genes in different developmental stages and the larval tissues of *P. xylostella*

**FIGURE 2:**
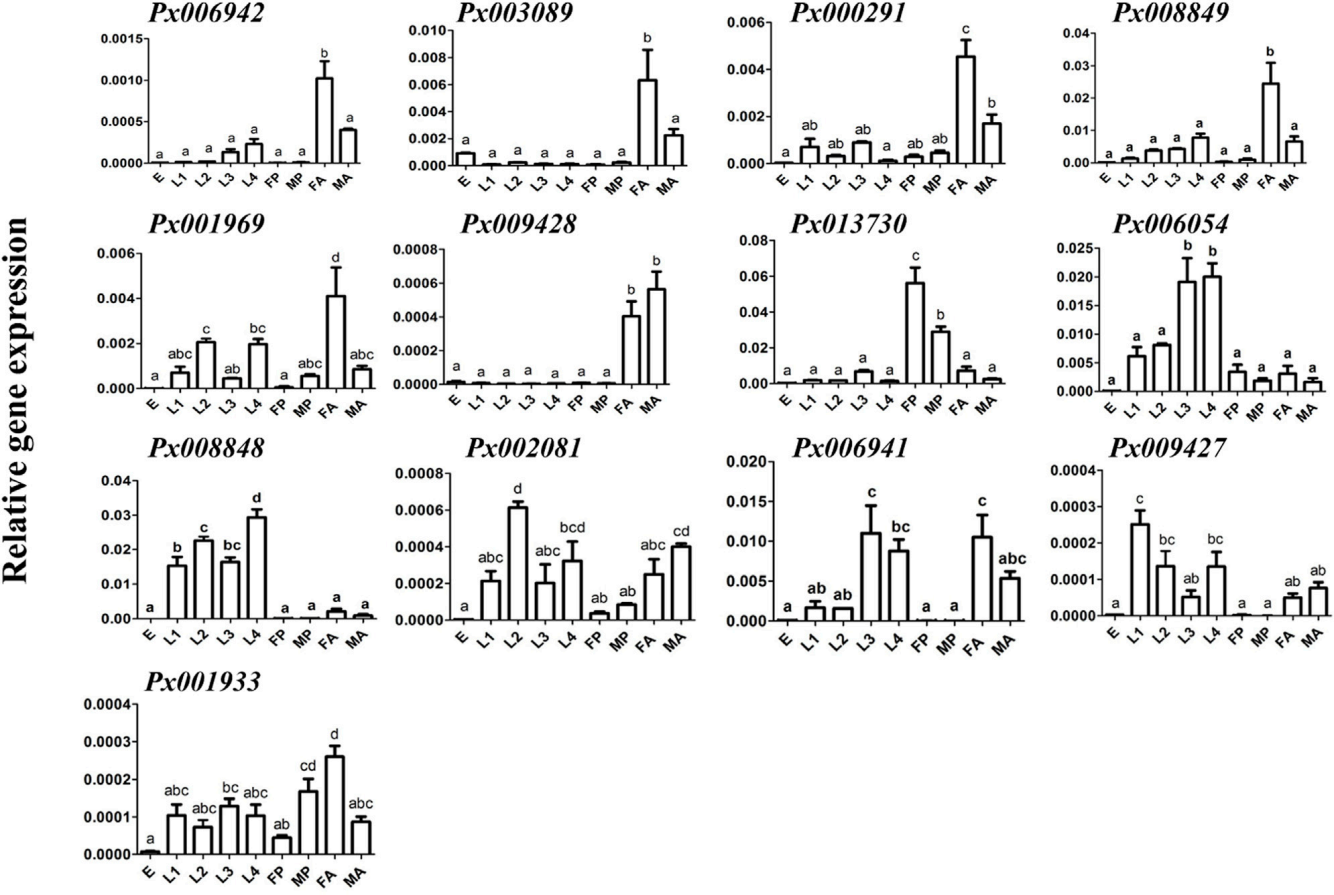
Expression profiling of GH1 genes at different developmental stages. Gene expressions of 13 GH1 genes at different developmental stages of *P. xylostella* in the AD strain were investigated using qRT-PCR. The data are shown as the mean ± standard error (SE), and different lowercase letters present a significant difference at the level of *p* ≤ 0.05 (n = 3). E: eggs; L1-L4: first - fourth instar larvae; FP and MP: mature female and male pupae; FA and MA: mature female and male adults.

**FIGURE 3:**
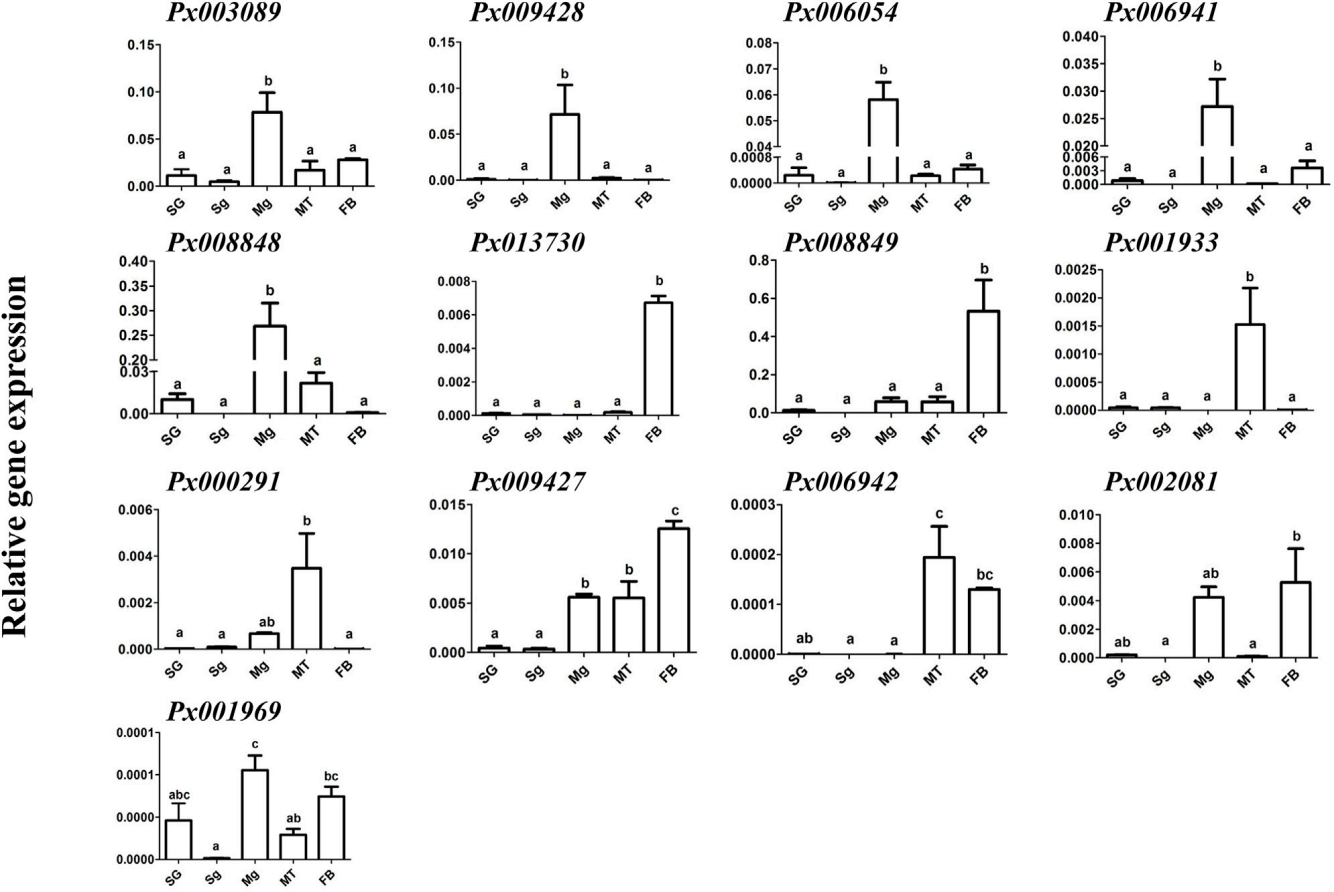
Expression profiling of GH1 genes in different tissues of the fourth instar larvae. Gene expression of 13 GH1 genes in different tissues dissected from fourth instar larvae in the AD strain was investigated using qRT-PCR. The data are shown as the mean ± SE, and different lowercase letters present a significant difference at the level of *p* ≤ 0.05 (n = 3). SG: salivary glands; Sg: silk glands; Mg: midgut; MT: Malpighian tubules; FB: fat body.

To further characterize the function of 13 putative GH1 genes, qRT-PCR was conducted to detect the expression of these genes at different developmental stages and larval tissues (Figure 2). We found that all of them were barely expressed in the eggs. The larva-specific candidate genes were *Px008848* and *Px006054,* with the former highly expressed throughout the larval stage and *Px006054* at the third and fourth instar larvae. Only *Px013730* was found to be expressed in the pupae, while six genes, *Px006942*, *Px003089*, *Px009169*, *Px008849*, *Px000291* and *Px009428,* were expressed in the adults. Expression of the remaining four putative GH1 genes showed no obvious spatiotemporal preference. Further expression profiling in tissues of the fourth instar larvae (Figure 3) identified five midgut-expressed genes (*Px003089*, *Px009428*, *Px006941*, *Px006054* and *Px008848*), two fat body-specific genes (*Px013730* and *Px008849*) and one Malpighian tubule-specific gene (*Px001933*). Expression of the remaining five putative GH1 genes could be detected at relatively high levels in more than two tissues.

### Selection of a candidate glycoside hydrolase family 1 gene involved in the interaction between the host plant and *P. xylostella*

**FIGURE 4:**
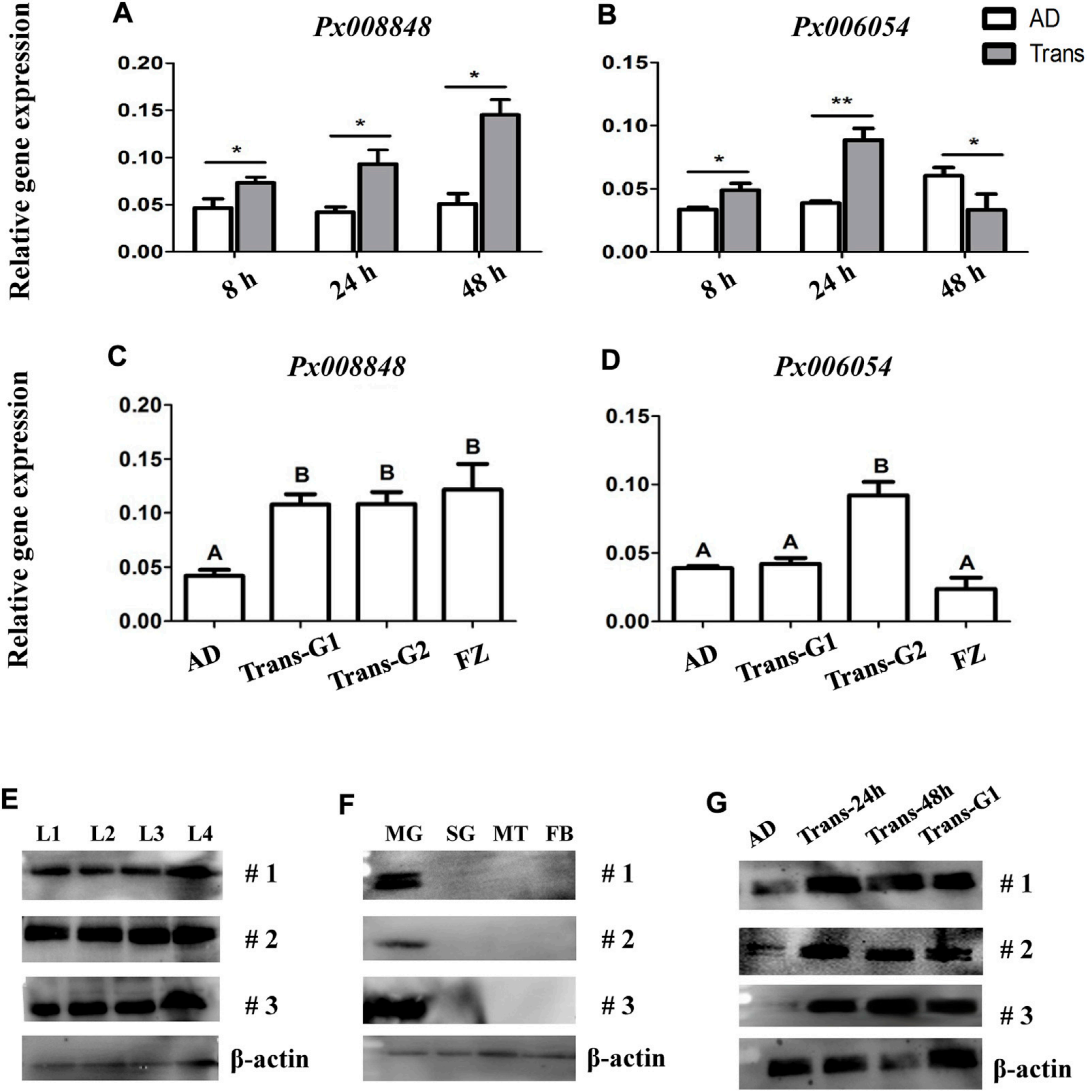
Expression profiling of *Px008848* and *Px006054* to host transfer. **(A)** and **(B)** The expression patterns of *Px008848* and *Px006054*, respectively, in the larval midgut of the AD strain feeding on artificial diet (AD) and after transfer onto radish cotyledons (Trans) in a short-term time course, including 8, 24 and 48 h. Student’s *t*-test was used for the analysis of gene expression between larvae feeding on an artificial diet and radish cotyledons. **(C)** and **(D)** The expression patterns of *Px008848* and *Px006054*, respectively, in the larval midgut of the AD strain after transfer from an artificial diet onto radish cotyledons over a long-term time course, including one (Trans-G1) and two generations (Trans-G2). The larvae of the AD and FZ strains (FZ) reared on artificial diets and radish cotyledons were used for comparison. One-way ANOVA followed by Tukey’s HSD post hoc test or student *t* test were used for the significant difference analysis of data (*p* ≤ 0.05, n = 3). **(E–F)** Protein expression profiling of Px008848 in larvae of different developmental stages **(E)**, different larval tissues **(F)** and larval midgut under plant challenge **(G)**. L1-L4: first–fourth instar larvae; MG: midgut; SG: salivary glands; MT: Malpighian tubules; FB: fat body; ^#^1–3: biological repeats 1–3. β-actin was used as a reference protein.

Since *Px008848* and *Px006054* were considered as larva-specific candidate genes, we further tested their expression patterns in AD larvae after short-term and long-term transfer from an artificial diet, which contained no chemical defense from the host plants, to the host plant radish. Compared with *Px006054*, the expression of *Px008848* in the larval midgut was consistently upregulated after host transfer from 8 h to 48 h and at generations G1 and G2 (Figures 4A–D). These patterns of *Px008848* further conformed to the expression profiling at the protein level using Western blotting, showing larval-midgut specificity and inducibility when larvae were fed with radish. (Figures 4E–G). Besides, *Px008848* was also found to be inducible when larvae were challenged by *A. thaliana* plant (Supplementary Figure S4). The results indicated that *Px008848* might be involved in the processes of the interaction between *P. xylostella* and the host plants.

### Prokaryotic expression and enzyme activity detection of Px008848

**FIGURE 5:**
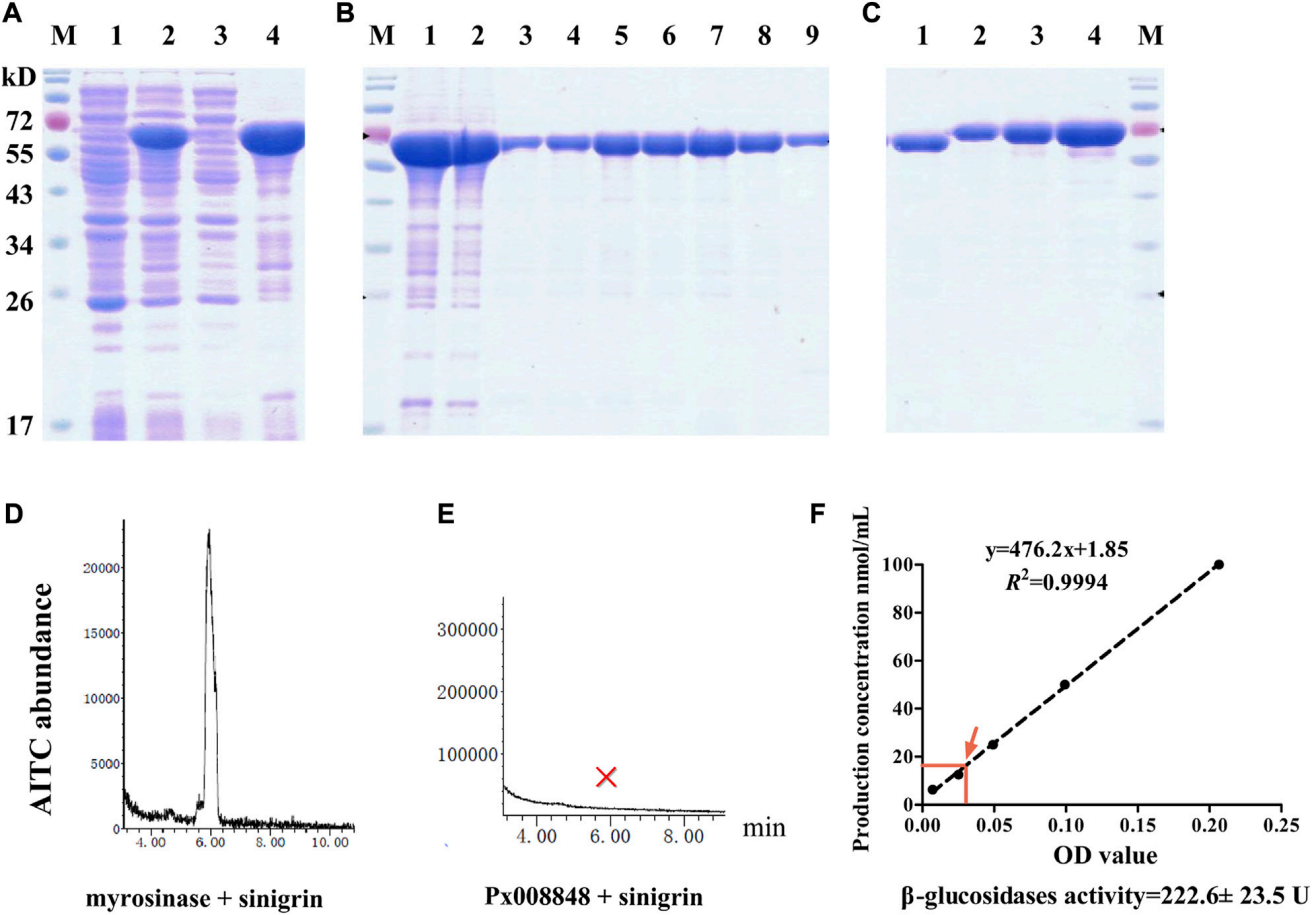
Prokaryotic expression and myrosinase activity of Px008848. **(A)** Electrophoretic diagram of competent cells with recombinant plasmid disrupted by sonication. Lanes 1 and 2: lysates of competent cells with recombinant plasmid treated with 0 or 0.5 mM inductive agent of IPTG, respectively; Lanes 3 and 4: supernatant and pellets of lysates disrupted by sonication. **(B)** Electrophoretic diagram of proteins treated with a Ni-NTA purification column. Lane 1: pellets of lysates disrupted by sonication; Lane 2: flow through purification column; Lanes 3–9: Px008848 protein eluted by 5, 10, 20, 30, 40, 50 or 200 mM elution buffer. **(C)** The verification and quantification of refolding Px008848 by using SDS-PAGE electrophoresis. Lane 1: 1 µL of Px008848 protein. Lanes 2–4: 1, 2 and 4 µg of bovine serum albumin standard protein. **(D)** and **(E)** The abundance of AITC produced by commercial myrosinase and purified Px008848 protein (20 µg) mixing with substrate sinigrin (10 mM), respectively. M: protein marker. **(F)** Detection of β-glucosidase activity of purified Px008848 protein. Standard curve was established based on the OD values of different concentrations of product p-nitrophenol. The mean OD value and the corresponding p-nitrophenol concentration produced by 10 µg purified Px008848 protein were marked with red arrow.

To determine enzyme activity of Px008848, the recombinant plasmid with *Px008848* was transformed into *E. coli* for prokaryotic protein expression. A clear band with a molecular weight of approximately 70 kD was found in IPTG-induced lysates and the corresponding pellets, which were detected by SDS-PAGE (Figure 5A). The IPTG-induced lysate was further loaded into a Ni-NTA purification column with different concentrations of imidazole to obtain recombinant Px008848 protein (Figure 5B). Moreover, although heterogenous expression of Px008848 in *E. coli* was incapable of glycosylation and it was unknown how much the protein glycosylation may influence the activity of Px008848, one *N*-glycosylation and three GalNAc-type *O*-glycosylation sites were potentially predicated in its deduced protein sequence (Supplementary Figure S5).

Based on an established system for detecting myrosinase activity by measuring the production of AITC, the protein of Px008848 after renaturation was quantified (Figure 5C) and incubated with sinigrin. The AITC signal was detected as expected from the combination of commercial plant myrosinase mixed with substrate sinigrin. However, no AITC signal was detected in the mixture of Px008848 and sinigrin (Figures 5D,E), indicating that the recombinant Px008848 protein barely possessed functional myrosinase activity. For the detection of β-glucosidase activity, the substrate of p-nitrophenyl β-d-glucopyranoside was incubated with purified Px008848 protein. Based on the standard curve, the β-glucosidase activity of Px008848 was verified and measured to be 222.6 ± 23.5 U (Figure 5F).

### Functional validation of Px008848 in insect-plant interactions

**FIGURE 6:**
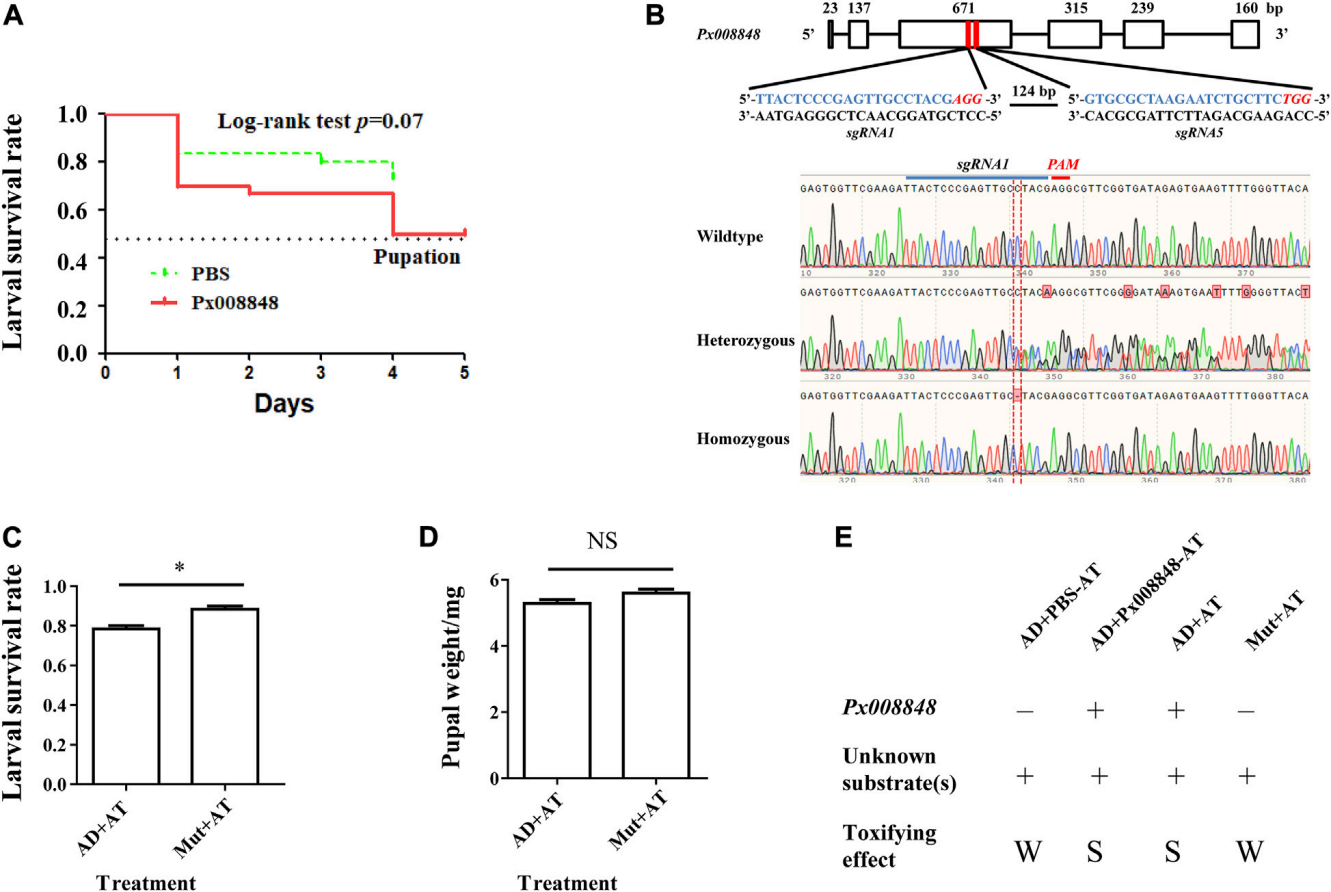
Effect of Px008848 on larval survival in *P. xylostella* under a host plant context. **(A)** Effect of wounded *A. thaliana* leaves treated with Px008848 on larval survival of AD strain. PBS: wounded *A. thaliana* leaves treated with 1 × PBS. Px008848: wounded *A. thaliana* leaves treated with 2 mg/ml Px008848. The larval survival rate (from larvae to pupae) was daily recorded when feeding on *A. thaliana* with different treatments. **(B)** Schematic diagram depicting the gene structure of *Px008848* and associated sgRNAs. Rectangular boxes denote six exons, and the numbers above show the length of the corresponding exons. Two sgRNAs were designed to target the third exon with a 124 bp interval. The sequences of the sgRNAs are marked in blue, and the sequences of the protospacer adjacent motifs (PAM, recognition site required for cleavage by Cas9 protein) are highlighted in italics and red. CRISPR/Cas9-mediated sequence-specific mutation in *Px008848* was verified by Sanger sequencing and the position of the 1 bp deletion is marked with a red vertical dotted line. **(C)** Larval survival rate and **(D)** pupal weight of the wild-type and mutant strains of *P. xylostella* reared on wild-type *A. thaliana* were compared. **(E)** Action mode of *Px008848.* AD: wild-type *P. xylostella* of the AD strain; Mut: *Px008848* mutant of the AD strain; AT: wild-type *A. thaliana*. *: *p* ≤ 0.05 (n = 3). NS: not significant. +: presence; −: absence; W: weak, S: strong.

Because the expression of *Px008848* was induced by the host plant, implying a potential role of *Px008848* in insect-plant interactions, the *A. thaliana* plants with wounded leaves under pretreatment of purified protein (2 mg/ml) or PBS for 15 min were renewed daily to feed *P. xylostella*. A reduced larval survival rate was observed for the larvae feeding on wounded *A. thaliana* leaves treated with Px008848 protein (Figure 6A), although the difference was not significant (*p* = 0.07).

To explore the function of Px008848 in the interaction between *P. xylostella* and *A. thaliana*, CRISPR/Cas9-based gene knockout technology was used to establish the homozygous mutant insect strain *Px008848*. About 200 freshly laid eggs of AD strain were micro-injected with sgRNAs-Cas9 complex. A homozygous mutant strain with a 1-bp deletion of *Px008848* was established, which was predicted to result in a frameshift mutation of the translated protein (Figure 6B). No obvious phenotypic changes were found between the wild-type and mutant strains according to daily observation. When the wild-type and mutant larvae were transferred onto the host *Arabidopsis*, the larval survival rate of the mutant larvae was significantly higher compared to that of the wild-type individuals (Figure 6C). However, no obvious change in pupal weight was found (Figure 6D).

## Discussion

In this study, we provided experimental evidence that no myrosinase activity could be detected in *P. xylostella*, although a total of 13 putative GH1 members were originally annotated as myrosinase-like genes in its genome (You et al., 2013). The expression level of a midgut-specific GH1 gene (*Px008848*) was upregulated in response to host plant challenge. The β-glucosidase activity and no myrosinase activity was detected from *in vitro* expressed Px008848, indicating that *Px008848* may participate in hydrolyzing glycoside substrate(s) other than glucosinolates. Based on biological assays of the mutant strain of *P. xylostella* feeding on *A. thaliana* Col-0 ecotype, we further confirmed that *Px008848* is involved in insect-plant interactions, although the specific substrate(s) in plants remains unclear.

Based on the GC-MS results, the myrosinase activity detected in the FZ larvae reared on host plant was derived from the plant debris in the larval digestive tract. Further, no myrosinase activity was detected in other developmental stages and larval tissues, suggesting that *P. xylostella* may not have functional myrosinases and these genes probably function in the metabolism of other substrates rather than GSs. While putative genes with sequence similarity to known plant or insect myrosinase genes could be found in insects, their functions should be verified by biochemical approaches. For example, although two glutamic acids are responsible for nucleophile and general acid/base catalyst groups in β-glucosidases, they also can be found in insect myrosinases with functional verification (Jones et al., 2002; Husebye et al., 2005). Meanwhile, the second glutamic acid is replaced with glutamine in myrosinases of *A. thaliana* (Burmeister et al., 2000). Phylogenetic analysis based on the sequences of functional insect myrosinases and other associated insect GH1 enzymes already revealed that insect myrosinases may have evolved in a species-specific way (Jones et al., 2002; Beran et al., 2014). Nonetheless, the potential effect of non-protein messenger/activator/ligand on myrosinase detection should also be considered, such as vitamin C is significantly related with up-regulation of the myrosinase activity (Ettlinger et al., 1961). The reason for the absence of myrosinase in *P. xylostella* may lie in that the GSs substrates are quickly depleted by GSSs in larval gut.

The expression profiling of 13 putative GH1 genes of *P. xylostella* showed specificity in both the developmental stages and larval tissues, implying the functional versatility of these genes (Mckenna et al., 2016). Among the 13 putative GH1 genes, the expression patterns of *Px008848* and *Px006054* were both predominantly larval- and midgut-specific. Notably, *Px008848* could respond to host plant challenge at both the RNA and protein levels, indicating that *Px008848* might be a potential candidate involved in the hydrolysis of plant glycosides. These data revealed that these putative GH1 genes might play diverse roles throughout the life cycle of *P. xylostella* and be important in midgut-related biological functions. To verify the characteristics of the enzyme activity of Px008848, a fusion protein with a histidine tag was heterogeneously expressed and was then incubated with substrate sinigrin to detect the production of AITC using GC-MS. Sinigrin has been extensively used as the testing substrate of myrosinase activity since it has a high affinity for the native myrosinase or the myrosinase produced in prokaryote (Jones et al., 2002; Li and Kushad., 2005). However, no AITC signal could be detected to prove the myrosinase activity of recombinant Px008848 protein, indicating that glucosinolate sinigrin was not the substrate of Px008848. Although natural insect myrosinase in *B. brassicae* is a dimer (Francis et al., 2002) and potential glycosylation sites were predicted in deduced protein sequence of Px008848, myrosinases from *P. striolata* and *B. brassicae* expressed *in vitro*, regardless of eukaryotic or prokaryotic expression system (Husebye et al., 2005; Beran et al., 2014), still showed physiochemical properties similar to those of the native enzyme for sinigrin. In addition, four myrosinase homologous genes of *P. striolata* also do not have myrosinase activities (Beran et al., 2014). Therefore, we concluded that *Px008848* is a GH1 member without myrosinase activity.

In addition to myrosinase, β-glucosidases are the most common members in the GH1 family and they are also involved in insect-plant interactions (Ketudat-Cairns & Esen, 2010). Although the β-glucosidase activity of Px008848 was verified, its optimum substrate and functions in insect-plant interaction are still unclear. Both of the plant- and insect-derived β-glucosidases can be directly responsible for the formation of aglycones from cyanogenic or iridoid glucosides (Zagrobelny et al., 2008; Dobler et al., 2011). Meanwhile, it has been identified in the large white butterfly *Pieris brassicae* that the caterpillar gut regurgitant secretes an elicitor β-glucosidase (Mattiacci et al., 1995), which induces the emission of volatiles of damaged cabbage plants and indirectly attracts the parasitic wasp *Cotesia glomerata*. This is similar to the finding for a piercing-sucking insect pest, rice brown planthopper *Nilaparvata lugens*, that salivary β-glucosidase-induced volatiles are also attractive to the parasitoid *Anagrus nilaparvatae* (Wang et al., 2008). Moreover, a β-glucosidase in midgut of the lepidopteran herbivore *M. sexta* is responsible for detoxifying lyciumoside IV, a major phytotoxin of its host plant *Nicotiana attenuata*, by catalyzing deglycosylation (Poreddy et al., 2015). A broad-spectrum β-glucosidase, Mm_bGlc17, in the larval gut of cockchafers (*M. melolontha*) is capable of reducing the toxicity of taraxinic acid β-d-glucopyranosyl ester from the host plant dandelion (*Taraxacum officinale*) through deglycosylation (Huber et al., 2021).

Interestingly, we found that the larval survival rate of *P. xylostella* was significantly impaired when the food contained Px008848 protein, indicating that this inducible gene resulted in negative effects when feeding on host plants. Therefore, we propose a possible two-component action mode to explain how *Px008848* functions during *P. xylostella* feeding on host plants, where one or more unknown chemical precursor(s) from cruciferous plants could be activated by protein Px008848 to deter *P. xylostella* (Figure 6E). It is possible that insect herbivores may possess a kind of β-glucosidases that are important for normal growth and development, while some of them unconsciously metabolize unknown plant-derived precursor(s) into toxic product upon feeding. However, this negative effect could hardly be detected and perhaps has been overcome in the natural population during the long-term interaction with host plants. Although accumulating cases of two-component cruciferous plant defense have been recorded, a two-component toxifying system containing insect-derived protein and plant-derived chemical compound(s) is rare (Pentzold et al., 2014). When plant tissues are consumed by insects, some plant-derived enzymes are suppressed to relatively low activities in the insect gut, such as the inhibition of hydrolysis against defensive salicin by β-glucosidases of *Salix* species under the alkaline environment of the gut lumen (Ruuhola et al., 2001; Ruuhola et al., 2003). In contrast, an insect-derived enzyme that is alkaline-insensitive might promote the efficacy of a two-component plant defense. However, in our case, further work is required to identify the plant-derived chemical substrate(s) of Px008848 and the metabolic pathway.

## Conclusion

Taken together, the identification and functional characterization of 13 putative GH1 genes revealed their multiple roles in a variety of physiological processes. More importantly, we found that one candidate, *Px008848*, seems to negatively affect the survival of *P. xylostella* when it feeds the host plant, although the underlying mechanism remains unclear. Our work provides evidence for the functional complexity of GH1 genes in insect-plant interactions, which is helpful for us to develop novel insecticide-free pest control strategies by exploring plant-derived chemical(s) to activate the toxifying effects of the insect GH1 genes.

## Data Availability

The original contributions presented in the study are included in the article/Supplementary Material, further inquiries can be directed to the corresponding authors.

## References

[B1] Ballhorn D. J. Kautz S. Lieberei R. (2010). Comparing responses of generalist and specialist herbivores to various cyanogenic plant features. Entomol. Exp. Appl. 134, 245–259. 10.1111/j.1570-7458.2009.00961.x 10.1111/j.1570-7458.2009.00961.x | Google Scholar

[B3] Beran F. Pauchet Y. Kunert G. Reichelt M. Wielsch N. Vogel H. (2014). *Phyllotreta striolata* flea beetles use host plant defense compounds to create their own glucosinolate-myrosinase system. Proc. Natl. Acad. Sci. U. S. A. 111, 7349–7354. 10.1073/pnas.1321781111 PubMed Abstract | 10.1073/pnas.1321781111 | Google Scholar 24799680 PMC4034198

[B4] Bones A. M. Rossiter J. T. (2010). The myrosinase-glucosinolate system, its organisation and biochemistry. Physiol. Plant. 97, 194–208. 10.1034/j.1399-3054.1996.970128.x 10.1034/j.1399-3054.1996.970128.x | Google Scholar

[B5] Bridges M. Jones A. M. Bones A. M. Hodgson C. Cole R. Bartlet E. (2002). Spatial organization of the glucosinolate-myrosinase system in brassica specialist aphids is similar to that of the host plant. Proc. Biol. Sci. 269, 187–191. 10.1098/rspb.2001.1861 PubMed Abstract | 10.1098/rspb.2001.1861 | Google Scholar 11798435 PMC1690872

[B6] Burmeister W. P. Cottaz S. Rollin P. Vasella A. Henrissat B. (2000). High resolution X-ray crystallography shows that ascorbate is a cofactor for myrosinase and substitutes for the function of the catalytic base. J. Biol. Chem. 275, 39385–39393. 10.1074/jbc.M006796200 PubMed Abstract | 10.1074/jbc.M006796200 | Google Scholar 10978344

[B7] Cantarel B. L. Coutinho P. M. Rancurel C. Bernard T. Lombard V. Henrissat B. (2009). The Carbohydrate-Active EnZymes database (CAZy): An expert resource for glycogenomics. Nucleic Acids Res. 37, 233–238. 10.1093/nar/gkn663 PubMed Abstract | 10.1093/nar/gkn663 | Google Scholar PMC2686590 18838391

[B8] Chen W. Dong Y. H. Saqib H. S. A. Vasseur L. Zhou W. W. Zheng L. (2020). Functions of duplicated glucosinolate sulfatases in the development and host adaptation of *Plutella xylostella* . Insect biochem. Mol. Biol. 119, 103316. 10.1016/j.ibmb.2020.103316 PubMed Abstract | 10.1016/j.ibmb.2020.103316 | Google Scholar 31953191

[B9] Chen W. Dong Y. Lin L. Saqib H. S. A. Ma X. Xu X. (2019). Implication for DNA methylation involved in the host transfer of diamondback moth, *Plutella xylostella* (L.). Arch. Insect Biochem. Physiol. 102, e21600. 10.1002/arch.21600 PubMed Abstract | 10.1002/arch.21600 | Google Scholar 31328824

[B10] Chen W. Saqib H. S. A. Xu X. Dong Y. Zheng L. Lai Y. (2022). Glucosinolate sulfatases–sulfatase-modifying factors system enables a crucifer-specialized moth to pre-detoxify defensive glucosinolate of the host plant. J. Agric. Food Chem. 70, 11179–11191. 10.1021/acs.jafc.2c03929 PubMed Abstract | 10.1021/acs.jafc.2c03929 | Google Scholar 36043275

[B11] Davies G. Henrissat B. (1995). Structures and mechanisms of glycosyl hydrolases. Structure 3, 853–859. 10.1016/s0969-2126(01)00220-9 PubMed Abstract | 10.1016/s0969-2126(01)00220-9 | Google Scholar 8535779

[B12] Desroches P. El-Shazly E. Mandon N. Duc G. Huignard J. (1995). Development of *Callosobruchus chinensis* (L.) and *C. maculatus* (F.) (Coleoptera: Bruchidae) in seeds of *Vicia faba* L. differing in their tannin, vicine and convicine contents. J. Stored Prod. Res. 31, 83–89. 10.1016/0022-474x(94)00028-r 10.1016/0022-474x(94)00028-r | Google Scholar

[B13] Desroches P. Mandon N. Baehr J. C. Huignard J. (1997). Mediation of host-plant use by a glucoside in *Callosobruchus maculatus* F. (Coleoptera: Bruchidae). J. Insect Physiol. 43, 439–446. 10.1016/s0022-1910(96)00123-0 10.1016/s0022-1910(96)00123-0 | Google Scholar

[B14] Dixon D. P. Sellars J. D. Kenwright A. M. Steel P. G. (2012). The maize benzoxazinone DIMBOA reacts with glutathione and other thiols to form spirocyclic adducts. Phytochemistry 77, 171–178. 10.1016/j.phytochem.2012.01.01910.1016/j.cois.2015.11.005 PubMed Abstract | 10.1016/j.phytochem.2012.01.01910.1016/j.cois.2015.11.005 | Google Scholar 22342783

[B15] Dobler S. Petschenka G. Pankoke H. (2011). Coping with toxic plant compounds–the insect’s perspective on iridoid glycosides and cardenolides. Phytochemistry 72, 1593–1604. 10.1016/j.phytochem.2011.04.015 PubMed Abstract | 10.1016/j.phytochem.2011.04.015 | Google Scholar 21620425

[B16] Erb M. Robert C. A. M. (2016). Sequestration of plant secondary metabolites by insect herbivores: Molecular mechanisms and ecological consequences. Curr. Opin. Insect Sci. 14, 8–11. 10.1016/j.cois.2015.11.005 PubMed Abstract | 10.1016/j.cois.2015.11.005 | Google Scholar 27436640

[B17] Ettlinger M. Dateo G. P. Harrison B. Mabry T. J. Thompson C. P. (1961). Vitamin C as a coenzyme: The hydrolysis of mustard oil glucosides. Proc. Natl. Acad. Sci. U. S. A. 47, 1875–1880. 10.1073/pnas.47.12.1875 PubMed Abstract | 10.1073/pnas.47.12.1875 | Google Scholar 13890918 PMC223232

[B18] Fahey J. W. Zalcmann A. T. Talalay P. (2001). The chemical diversity and distribution of glucosinolates and isothiocyanates among plants. Phytochemistry 56, 5–51. 10.1016/s0031-9422(00)00316-2 PubMed Abstract | 10.1016/s0031-9422(00)00316-2 | Google Scholar 11198818

[B19] Francis F. Lognay G. Wathelet J. P. Haubruge E. (2002). Characterisation of aphid myrosinase and degradation studies of glucosinolates. Arch. Insect Biochem. Physiol. 50, 173–182. 10.1002/arch.10042 PubMed Abstract | 10.1002/arch.10042 | Google Scholar 12125058

[B21] Halkier B. A. Gershenzon J. (2006). Biology and biochemistry of glucosinolates. Annu. Rev. Plant Biol. 57, 303–333. 10.1146/annurev.arplant.57.032905.105228 PubMed Abstract | 10.1146/annurev.arplant.57.032905.105228 | Google Scholar 16669764

[B22] Heidel-Fischer H. M. Kirsch R. Reichelt M. Ahn S. J. Wielsch N. Baxter S. W. (2019). An insect counteradaptation against host plant defenses evolved through concerted neofunctionalization. Mol. Biol. Evol. 36, 930–941. 10.1093/molbev/msz019 PubMed Abstract | 10.1093/molbev/msz019 | Google Scholar 30715408 PMC6501874

[B23] Huang Y. Wang Y. Zeng B. Liu Z. Xu X. Meng Q. (2017). Functional characterization of Pol III U6 promoters for gene knockdown and knockout in *Plutella xylostella* . Insect biochem. Mol. Biol. 89, 71–78. 10.1016/j.ibmb.2017.08.009 PubMed Abstract | 10.1016/j.ibmb.2017.08.009 | Google Scholar 28890398

[B24] Huber M. Roder T. Irmisch S. Riedel A. Gablenz S. Fricke J. (2021). A beta-glucosidase of an insect herbivore determines both toxicity and deterrence of a dandelion defense metabolite. eLife 10, e68642. 10.7554/eLife.68642 PubMed Abstract | 10.7554/eLife.68642 | Google Scholar 34632981 PMC8504966

[B25] Husebye H. Arzt S. Burmeister W. P. Hartel F. V. Brandt A. Rossiter J. T. (2005). Crystal structure at 1.1 Angstroms resolution of an insect myrosinase from *Brevicoryne brassicae* shows its close relationship to beta-glucosidases. Insect biochem. Mol. Biol. 35, 1311–1320. 10.1016/j.ibmb.2005.07.004 PubMed Abstract | 10.1016/j.ibmb.2005.07.004 | Google Scholar 16291087

[B26] Jones A. M. E. Winge P. Bones A. M. Cole R. Rossiter J. T. (2002). Characterization and evolution of a myrosinase from the cabbage aphid *Brevicoryne brassicae* . Insect biochem. Mol. Biol. 32, 275–284. 10.1016/s0965-1748(01)00088-1 PubMed Abstract | 10.1016/s0965-1748(01)00088-1 | Google Scholar 11804799

[B27] Kazana E. Pope T. W. Tibbles L. Bridges M. Pickett J. A. Bones A. M. (2007). The cabbage aphid: A walking mustard oil bomb. Proc. Biol. Sci. 274, 2271–2277. 10.1098/rspb.2007.0237 PubMed Abstract | 10.1098/rspb.2007.0237 | Google Scholar 17623639 PMC2288485

[B28] Ketudat-Cairns J. R. Esen A. (2010). β-Glucosidases. Cell. Mol. Life Sci. 67, 3389–3405. 10.1007/s00018-010-0399-2 PubMed Abstract | 10.1007/s00018-010-0399-2 | Google Scholar 20490603 PMC11115901

[B29] Kissen R. Rossiter J. T. Bones A. M. (2009). The ‘mustard oil bomb’: Not so easy to assemble?! Localization, expression and distribution of the components of the myrosinase enzyme system. Phytochem. Rev. 8, 69–86. 10.1007/s11101-008-9109-1 10.1007/s11101-008-9109-1 | Google Scholar

[B30] Kliebenstein D. J. Kroymann J. Mitchell-Olds T. (2005). The glucosinolate–myrosinase system in an ecological and evolutionary context. Curr. Opin. Plant Biol. 8, 264–271. 10.1016/j.pbi.2005.03.002 PubMed Abstract | 10.1016/j.pbi.2005.03.002 | Google Scholar 15860423

[B31] Koroleva O. A. Davies A. Deeken R. Thorpe M. R. Tomos A. D. Hedrich R. (2000). Identification of a new glucosinolate-rich cell type in *Arabidopsis* flower stalk. Plant Physiol. 124, 599–608. 10.1104/pp.124.2.599 PubMed Abstract | 10.1104/pp.124.2.599 | Google Scholar 11027710 PMC59166

[B32] Li X. Kushad M. M. (2005). Purification and characterization of myrosinase from horseradish (*Armoracia rusticana*) roots. Plant Physiol. biochem. 43, 503–511. 10.1016/j.plaphy.2005.03.015 PubMed Abstract | 10.1016/j.plaphy.2005.03.015 | Google Scholar 15922609

[B33] Livak K. J. Schmittgen T. D. (2002). Analysis of relative gene expression data using real-time quantitative PCR and the 2(-Delta Delta C(T)) Method. Methods 25, 402–408. 10.1006/meth.2001.1262 PubMed Abstract | 10.1006/meth.2001.1262 | Google Scholar 11846609

[B34] Ma X. L. He W. Y. Chen W. Xu X. J. Qi W. P. Zou M. M. (2018). Structure and expression of sulfatase and sulfatase modifying factor genes in the diamondback moth, *Plutella xylostella* . Insect Sci. 25, 946–958. 10.1111/1744-7917.12487 PubMed Abstract | 10.1111/1744-7917.12487 | Google Scholar 28569426

[B35] Mattiacci L. Dicke M. Posthumus M. A. (1995). beta-Glucosidase: an elicitor of herbivore-induced plant odor that attracts host-searching parasitic wasps. Proc. Natl. Acad. Sci. U. S. A. 92, 2036–2040. 10.1073/pnas.92.6.2036 PubMed Abstract | 10.1073/pnas.92.6.2036 | Google Scholar 11607516 PMC42418

[B36] Mckenna D. D. Scully E. D. Pauchet Y. Hoover K. Kirsch R. Geib S. M. (2016). Genome of the Asian longhorned beetle (*Anoplophora glabripennis*), a globally significant invasive species, reveals key functional and evolutionary innovations at the beetle-plant interface. Genome Biol. 17, 227. 10.1186/s13059-016-1088-8 PubMed Abstract | 10.1186/s13059-016-1088-8 | Google Scholar 27832824 PMC5105290

[B37] Møller B. L. (2010). Functional diversifications of cyanogenic glucosides. Curr. Opin. Plant Biol. 13, 338–347. 10.1016/j.pbi.2010.01.009 PubMed Abstract | 10.1016/j.pbi.2010.01.009 | Google Scholar 20197238

[B38] Morant A. V. Jørgensen K. Jørgensen C. Paquette S. M. SánchezPérez R. Møller B. L. (2008). beta-Glucosidases as detonators of plant chemical defense. Phytochemistry 69, 1795–1813. 10.1016/j.phytochem.2008.03.006 PubMed Abstract | 10.1016/j.phytochem.2008.03.006 | Google Scholar 18472115

[B39] Müller C. Boeve J. L. Brakefield P. (2002). Host plant derived feeding deterrence towards ants in the turnip sawfly *Athalia rosae* . Entomol. Exp. Appl. 104, 153–157. 10.1046/j.1570-7458.2002.01002.x 10.1046/j.1570-7458.2002.01002.x | Google Scholar

[B40] Müller C. (2009). Interactions between glucosinolate- and myrosinase-containing plants and the sawfly *Athalia rosae* . Phytochem. Rev. 8, 121–134. 10.1007/s11101-008-9115-3 10.1007/s11101-008-9115-3 | Google Scholar

[B41] Müller C. Wittstock U. (2005). Uptake and turn-over of glucosinolates sequestered in the sawfly *Athalia rosae* . Insect biochem. Mol. Biol. 35, 1189–1198. 10.1016/j.ibmb.2005.06.001 PubMed Abstract | 10.1016/j.ibmb.2005.06.001 | Google Scholar 16102424

[B42] Opitz S. E. W. Müller C. (2009). Plant chemistry and insect sequestration. Chemoecology 19, 117–154. 10.1007/s00049-009-0018-6 10.1007/s00049-009-0018-6 | Google Scholar

[B43] Pankoke H. Bowers M. D. Dobler S. (2012). The interplay between toxinreleasing β-glucosidase and plant iridoid glycosides impairs larval development in a generalist caterpillar, *Grammia incorrupta* (Arctiidae). Insect biochem. Mol. Biol. 42, 426–434. 10.1016/j.ibmb.2012.02.004 PubMed Abstract | 10.1016/j.ibmb.2012.02.004 | Google Scholar 22446106

[B44] Pentzold S. Zagrobelny M. Rook F. Bak S. (2014). How insects overcome two-component plant chemical defence: Plant β-glucosidases as the main target for herbivore adaptation. Biol. Rev. Camb. Philos. Soc. 89, 531–551. 10.1111/brv.12066 PubMed Abstract | 10.1111/brv.12066 | Google Scholar 25165798

[B45] Poreddy S. Mitra S. Schottner M. Chandran J. Schneider B. Baldwin I. T. (2015). Detoxification of hostplant's chemical defence rather than its anti-predator co-option drives β-glucosidase-mediated lepidopteran counteradaptation. Nat. Commun. 6, 8525. 10.1038/ncomms9525 PubMed Abstract | 10.1038/ncomms9525 | Google Scholar 26443324 PMC4633822

[B46] Ratzka A. Vogel H. Kliebenstein D. J. Mitchell-Olds T. Kroymann J. (2002). Disarming the mustard oil bomb. Proc. Natl. Acad. Sci. U. S. A. 99, 11223–11228. 10.1073/pnas.17211289 PubMed Abstract | 10.1073/pnas.17211289 | Google Scholar 12161563 PMC123237

[B47] Robert C. A. M. Zhang X. Machado R. A. Schirmer S. Lori M. Mateo P. (2017). Sequestration and activation of plant toxins protect the Western corn rootworm from enemies at multiple trophic levels. eLife 6, 29307. 10.7554/eLife.29307 PubMed Abstract | 10.7554/eLife.29307 | Google Scholar PMC5701792 29171835

[B48] Ruuhola T. Julkunen-Tiitto R. Vainiotalo P. (2003). *In vitro* degradation of willow salicylates. J. Chem. Ecol. 29, 1083–1097. 10.1023/A:1023821304656 PubMed Abstract | 10.1023/A:1023821304656 | Google Scholar 12857023

[B49] Ruuhola T. Tikkanen O. P. Tahvanainen J. (2001). Differences in host use efficiency of larvae of a generalist moth, *Operophtera brumata* on three chemically divergent salix species. J. Chem. Ecol. 27, 1595–1615. 10.1023/A:1010458208335 PubMed Abstract | 10.1023/A:1010458208335 | Google Scholar 11521399

[B50] Rye C. S. Withers S. G. (2000). Glycosidase mechanisms. Curr. Opin. Chem. Biol. 4, 573–580. 10.1016/S1367-5931(00)00135-6 PubMed Abstract | 10.1016/S1367-5931(00)00135-6 | Google Scholar 11006547

[B51] Tang W. Yu L. He W. Yang G. Ke F. Baxter S. W. (2014). DBM-DB: The diamondback moth genome database. Database 2014, bat087. 10.1093/database/bat087 PubMed Abstract | 10.1093/database/bat087 | Google Scholar 24434032 PMC3893660

[B52] Textor S. Gershenzon J. (2009). Herbivore induction of the glucosinolate–myrosinase defense system: Major trends, biochemical bases and ecological significance. Phytochem. Rev. 8, 149–170. 10.1007/s11101-008-9117-1 10.1007/s11101-008-9117-1 | Google Scholar

[B53] Wang X. Zhou G. X. Xiang C. Y. Du M. H. Cheng J. A. Liu S. S. (2008). β-Glucosidase treatment and infestation by the rice Brown planthopper *Nilaparvata lugens* elicit similar signaling pathways in rice plants. Chin. Sci. Bull. 53, 53–57. 10.1007/s11434-008-0048-4 10.1007/s11434-008-0048-4 | Google Scholar

[B54] Yang Z. L. Nour-Eldin H. H. Hänniger S. Reichelt M. Crocoll C. Seitz F. (2021). Sugar transporters enable a leaf beetle to accumulate plant defense compounds. Nat. Commun. 12, 2658. 10.1038/s41467-021-22982-8 PubMed Abstract | 10.1038/s41467-021-22982-8 | Google Scholar 33976202 PMC8113468

[B55] You M. Yue Z. He W. Yang X. Yang G. Xie M. (2013). A heterozygous moth genome provides insights into herbivory and detoxification. Nat. Genet. 45, 220–225. 10.1038/ng.2524 PubMed Abstract | 10.1038/ng.2524 | Google Scholar 23313953

[B56] Zagrobelny M. Bak S. Ekstrøm C. T. Olsen C. E. Møller B. L. Lindberg Moller B. (2004). Cyanogenic glucosides and plant–insect interactions. Phytochemistry 65, 293–306. 10.1016/j.phytochem.2003.10.016 PubMed Abstract | 10.1016/j.phytochem.2003.10.016 | Google Scholar 14751300

[B57] Zagrobelny M. Bak S. Møller B. L. (2008). Cyanogenesis in plants and arthropods. Phytochemistry 69, 1457–1468. 10.1016/j.phytochem.2008.02.019 PubMed Abstract | 10.1016/j.phytochem.2008.02.019 | Google Scholar 18353406

[B58] Zalucki M. P. Shabbir A. Silva R. Adamson D. Liu S. S. Furlong M. J. (2012). Estimating the economic cost of one of the world's major insect pests, *Plutella xylostella* (Lepidoptera: Plutellidae): Just how long is a piece of string? J. Econ. Entomol. 105, 1115–1129. 10.1603/EC12107 PubMed Abstract | 10.1603/EC12107 | Google Scholar 22928287

