# Supplementary material for: An inducible gene from glycoside hydrolase one family of Plutella xylostella decreases larval survival when feeding on host plant
Source: Front Physiol. 2022 Oct 20;13:1013092. doi: 10.3389/fphys.2022.1013092 (PMC9632345; doi:10.3389/fphys.2022.1013092)
Supplement: Supplementary file 1 [file DataSheet2.PDF]

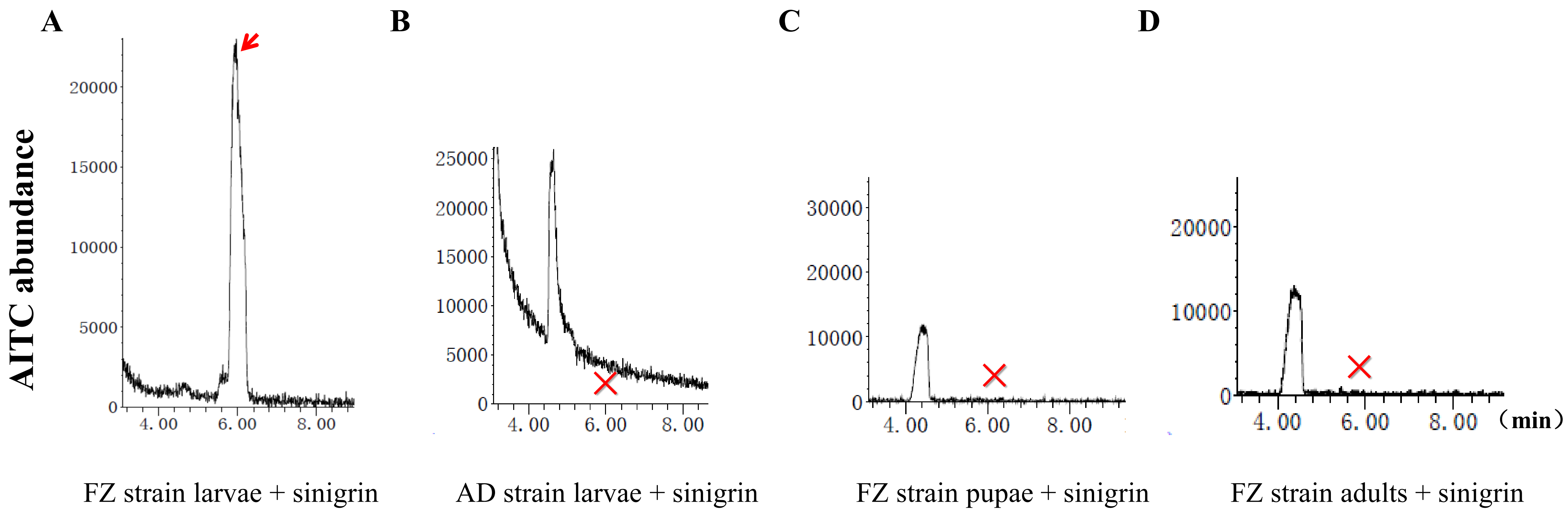
A
B
C
D
AITC abundance
20000
15000
10000
5000
0
25000
30000
4.00
6.00
8.00
（min）
FZ strain larvae + sinigrin
AD strain larvae + sinigrin
FZ strain pupae + sinigrin
FZ strain adults + sinigrin
