# Supplementary figures and images for: An inducible gene from glycoside hydrolase one family of Plutella xylostella decreases larval survival when feeding on host plant
Source: Front Physiol. 2022 Oct 20;13:1013092. doi: 10.3389/fphys.2022.1013092 (PMC9632345; doi:10.3389/fphys.2022.1013092)

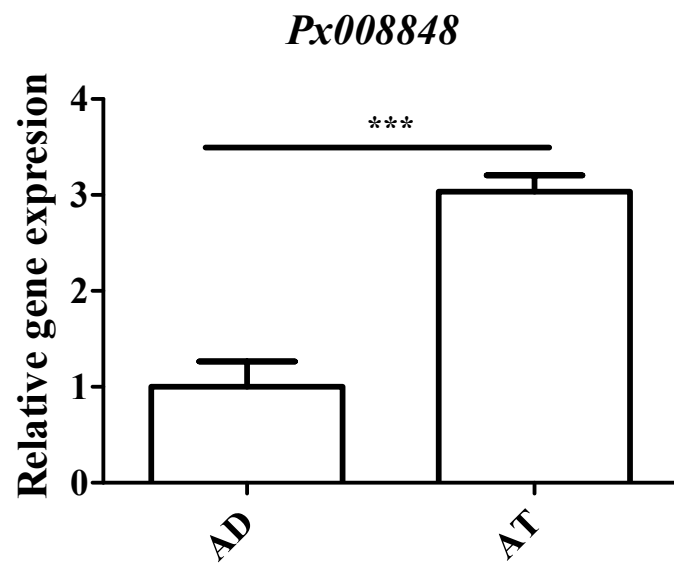

Supplement: Supplementary file 2 [file DataSheet4.PDF]
