# Supplementary material for: An inducible gene from glycoside hydrolase one family of Plutella xylostella decreases larval survival when feeding on host plant
Source: Front Physiol. 2022 Oct 20;13:1013092. doi: 10.3389/fphys.2022.1013092 (PMC9632345; doi:10.3389/fphys.2022.1013092)
Supplement: Supplementary file 3 [file Table1.DOCX]

**Table S1 The primers used in this study**

| Gene ID | Primer | | | | | | | | | Length (bp) |  |
| --- | --- | --- | --- | --- | --- | --- | --- | --- | --- | --- | --- |
|  | Sequence | | | | Tm (°C) | | | | |  |  |
| **Full-length amplification of CDS** | | | | | | | | | | | |
| *Px006941* | F：TAAATCTTTGACAAGTCCCCGA  R：TTGTTTATAATAAGGTAAGCAC | | | | | 55  48 | | | | 1494 |  |
| *Px008848* | F：CCTATATCTCTCGTCTCACC  R：TGGTATGATTGGTAGGTATG | | | | | 48  48 | | | | 1746 |  |
| *Px008849* | F：CCTGTAATAAAGAAGGCTAATC  R：ATCAAAAGGAAAGAAAAGCCC | | | | | 51  57 | | | | 1745 |  |
| *Px006954* | F：TCGCCAGTTCGTTGTTGATA  R：GTCTAAATATCTACAAAACACCGAAC | | | | | 57  55 | | | | 1657 |  |
| **Partial-length amplification of CDS** | | | | | | | | | | | |
| *Px009427*  *-part* | F：GTTTATGAGAGGGAGTTTAAGGGA  R：GCCGTGAACTGGTTGGAAG | | | | | 55  58 | | | | 550 |  |
| *Px009428*  *-part* | F：GTTTATGAGAGGGAGTTTAAGGGA  R：GCCGTGAACTGGTTGGAAG | | | | | 55  58 | | | | 368 |  |
| *Px000291*  *-part* | F：CCGCGGACTCGTACCACCGCT  R：CCGCGGACTCGTACCACCGCT | | | | | 68  69 | | | | 789 |  |
| **qRT-PCR** |  | | | |  | | | | |  |  |
| *Px008849* | | | | F: CTACTGGCTTCTCCAACTATA  R: TCACTATCGGCTCTATATTATACT | | | 60.2  59.8 | | | 106 |  |
| *Px008848* | | | | F: GATATTGATGCTGAGGCTATAC  R: TGTAGTAGACTTGGTCATTGTA | | | 60.1  60.2 | | | 142 |  |
| *Px006941* | | | | F: TGTGTGTCAAGAATGTGCTGAT  R: GCTTCATACCACATGAAATGATTTG | | | 61.2  58.4 | | | 112 |  |
| *Px009428* | | | | F: GTTTATGAGAGGGAGTTTAAGGGA  R: GGCTACTTCCAAATCCTTCTC | | | 61.8  62.0 | | | 107 |  |
| *Px006054* | | | | F: TCGCCAGTTCGTTGTTGATAGCC  R: TGGCAACTCCGAAAGTGAACCCT | | | 66.2  66.9 | | | 140 |  |
| *Px002081* | | | | F: ATGAAGGTTACGGCTCCACC  R: GTAGTTCTCGTCATACAAATGGTACAC | | | 64.0  61.8 | | | 235 |  |
| *Px000291* | | | | F: GCACGGGGCTCAGACGCTTA  R: GCCTCCTTCATTCCAAGCACCT | | | 65.7  64.4 | | | 123 |  |
| *Px001933* | | | | F: CTTGGTAGTGCCATACTGCTGT  R: ACCTCCTTCATTCCAAGCACCT | | | 57.3  61.7 | | | 168 |  |
| *Px003089* | | | | F: ATGCTAGAAATTTGCTTGAACGATA  R: TGACGCAAATATACTCAGCTATCC | | | 63.8  60.8 | | | 121 |  |
| *Px006942* | | | | F: TTTTGGCAGTTGGTTGTGTTCA  R: TTGTTGATTTATCACTCTGGTTCC | | | 61.4  58.4 | | | 151 |  |
| *Px001969* | | | | F: GCATACCAAGTGGAGGGCG  R: TGTGGTACGAGTCTGCCGAA | | | 61.1  59.6 | | | 130 |  |
| *Px009427* | | | | F: CCAAGCATATGGGACAAGGTG  R: AGTCAAGGCCCAATTCCTTGG | | | 59.0  59.6 | | | 162 |  |
| *Px013730* | | | | F: CGGTGTTTCTACATCGGCTTTC  R: GTTGGTGTGGTCGAATACGC | | | 61.5  58.5 | | | 118 |  |
| PCR production was confirmed by Sanger sequencing | | | | | | | | | | | |
| **Prokaryotic expression of *Px008848*** | | | | | | | | | | | |
| *Px008848*-expression-F | | | **^#^** F：AAGCTTCCTATATCTCTCGTCTCACC | | | | | |  |  |  |
| *Px008848*-expression-R | | | **^#^** R：CTCGAGTGGTATGATTGGTAGGTATG | | | | | |  |  |  |
| **#**: Sequences of restriction enzyme site of *BamH*I and *Xho*I were underlined. | | | | | | | | | | | |
| **Mutant strain of *Px008848*** | | | | | | | | | | | |
| CRISPR-F | | ******TAATACGACTCACTATAGG***N_20_**GTTTTAGAGCTAG | | | | | | |  |  |  |
| CRISPR-R | | AAAAGCACCGACTCGGTGCC | | | | | | |  |  |  |
| sgRNA-1 | | TTACTCCCGAGTTGCCTACG | | | | | | |  |  |  |
| sgRNA-5 | | GTGCGCTAAGAATCTGCTTC | | | | | | |  |  |  |
| *****: A T7 polymerase-binding site sequence was in italic, N_20_ (marked in bold) can be replaced by corresponding sequence of sgRNA-1 or sgRNA-5. Partial sequence of sgRNA scaffold plasmid was underlined. The whole sgRNA scaffold sequence was artificially synthetized and cloned to plasmid as a scaffold plasmid (sequence is same to GenBank ID: MG917725). | | | | | | | | | | | |
| **Mutant detection in *Px008848*** | | | | | | | | | | | |
| *Px008848*-test-F | | | GTAAAGGCGAAAACATTTGG | | | | |  | |  |  |
| *Px008848*-test-R | | | GTAGCCTGGTTATACAATTCACAAG | | | | |  | |  |  |
| **Transgenic *A. thaliana* over-expressing *Px008848*** | | | | | | | | | | | |
| *KnpI*-8848-F | | ¶ *GG*GGTACCCCTATATCTCTCGTCTCACC | | | | |  | | |  |  |
| *PstI*-8848-R | | ¶ *AA*CTGCAGTGGTATGATTGGTAGGTATG | | | | |  | | |  |  |
| ¶: The oligo sequences were marked in italic. Sequences of restriction enzyme site were underlined. | | | | | | | | | | | |
