# Supplementary material for: An inducible gene from glycoside hydrolase one family of Plutella xylostella decreases larval survival when feeding on host plant
Source: Front Physiol. 2022 Oct 20;13:1013092. doi: 10.3389/fphys.2022.1013092 (PMC9632345; doi:10.3389/fphys.2022.1013092)
Supplement: Supplementary file 4 [file DataSheet3.PDF]

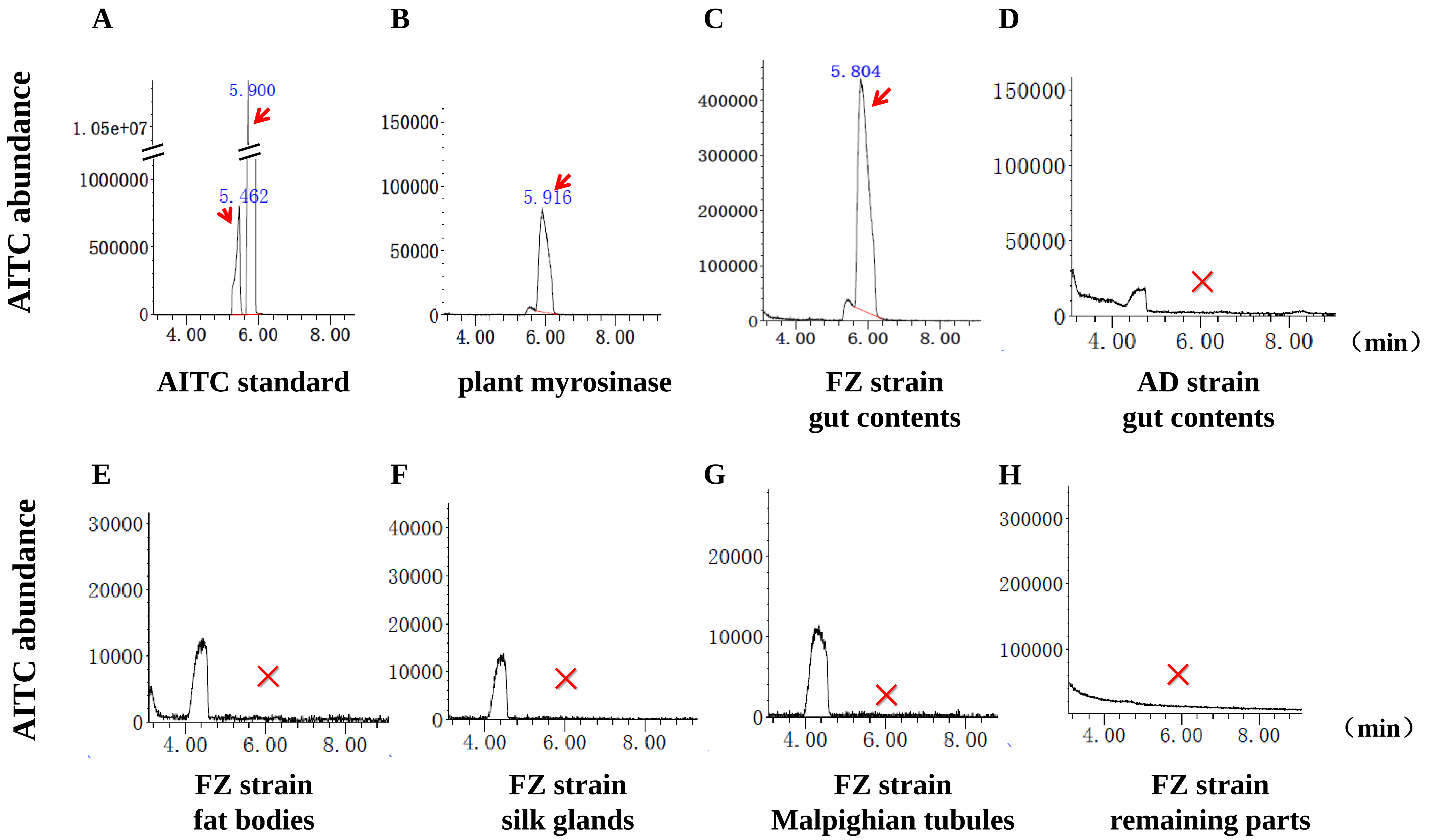

A
B
C
D
AITC abundance
1.05e+07
1000000
500000
0
5.900
5.462
4.00
6.00
8.00
AITC standard
150000
100000
50000
5.916
plant myrosinase
400000
300000
200000
100000
5.804
FZ strain
gut contents
AD strain
gut contents
(min)
E
F
G
H
30000
20000
10000
FZ strain
fat bodies
40000
FZ strain
silk glands
FZ strain
Malpighian tubules
300000
FZ strain
remaining parts
