# Supplementary material for: An inducible gene from glycoside hydrolase one family of Plutella xylostella decreases larval survival when feeding on host plant
Source: Front Physiol. 2022 Oct 20;13:1013092. doi: 10.3389/fphys.2022.1013092 (PMC9632345; doi:10.3389/fphys.2022.1013092)
Supplement: Supplementary file 5 [file Table2.DOCX]

**Table S2 PCR results of the GH1 genes in *P. xylostella***

| >*Px006941* full-length amplification in FZ strain  TAAATCTTTGACAAGTCCCCGAGCGTGTGGGACCACTTCATCCACTCGACCCCTGACCTGGTGGCGGACCGCTCCACCGGGGACGTGGCCGCAGACTCCTACCACTTGTGGAGACGAGACGTGGAGATGCTGGAGGAGCTAGGGCTGCAGTTTTATAGATTCTCCATATCCTGGACGCGTCTGGTACCGAATGGATTTGCGAACGTGATCAGCGATGACGGCCGGCGGTACTACGATGACCTCATCAACGGTCTGTTGGAGAGCGGAATAGAACCCATCCCCACCATCTATCATTGGGACATACCGCAACGGTTGCAGGATTTGGGTGGCTGGACCAACCCACTCGTATCAGACTGGTTTGTGGAATACGCAAGAATGGTCTTTTCCCTCTACGCTGACCGAGTCAAGTACTGGGTGACCATCAACGAGCCCTCAGTGTTCTGTGAGTTTGGATACGGAGCTGGTATTATAGCACCTGGGATCAAAGACCAGGATATCGGCAAGTACATGTGTGTCAAGAATGTGCTGATGGCCCATGGGAATGGGAAGGCGTATAGGGTTTATGAGAGGGAGTTTAAGGGAAAATATTCTGGAAAGCTATCGATGGCAAATCATTTCATGTGGTATGAAGCTGAGGCGGAGAAGGATTTGGAAGTAGCTGAACAAATAAGGCAGTTTCAGTACGGCCTGTACTGCCACGCCATCTACTCGGCGGCGGGCGGCTGGCCGGCGGCGGTGGAGCGGCAGATAGCTGACAACAGCCGCAGAGAGGGCTACAGCTTCTCCAGACTGCCGGGGTTCACGCAGGAGGAGAAGGACTTGGTCAGAGGTACCTTCGACTACATGGCAGTGAACCACTACAGCAGCAGGACAGTCCGGAAACAGCCGTCAGCGGAACACATCCCTGCTTCCAACCAGTTCACGGCGAATGCTGAGTTTAATGCCACGTATGAGGGTAAAGAGGACTGGCCTAAAGGAAACTCCTTTTGGTTCTTGATAAACCCGGCCGGCTTGCGTGACCTGCTCGTGTGGTTAAGGACAGAGTACGGGGACCTCGTGTACCTCTTCACAGAGAACGGGTACTCTGACGTCACCAGCAAGATCAATGATGACGATAGGGTTGACTATCATAGGGAGTATTTGAAACAGGTTCTTTTGGCCATAAAGCAAGACGGAGTGAATGTGACTGGCTACACGGCGTGGTCTCTGATGGACGACTTCGAGTGGGTGGAGGGATACACTCTCGGTTTCGGTCTATACCACGTGGACTTCAGCGGGGCGGAGCGCCCGCGCACGCCGCGCAAGTCCGCGCGCTACTACGCCAGCGTCGCGCGAGCCAACTCGCTGGACGTGCCAGTGCCTGAAGATGTCATGTTGAACGCAGCTTACTCGTTTGTTAACTCCACTTGCACTCTGATGGCCATGATCGTCGTGGTCGCATGTTACCGCATCTTCAACTTTTAATGAATAGGGTGCTTACCTTATTATAAACAA |
| --- |
| >*Px008848* full-length amplification in FZ strain  CCTATATCTCTCGTCTCACCAATAAACCATGACATTCAGCTTATCCTATCGTGTAATAGTCCTGGTTGCATTGCTAAGTGTAGCGTCGGGAAAAACAAAAGATGTGCACAGCAGAAAGTTTCCCGATGACTTTTTGTTTGGGGCAGCCACGGCGTCTTACCAGATAGAAGGCGCTTGGAATGAGGATGGTAAAGGCGAAAACATTTGGGATCGTTTGACTCACAGAGACCCTTCGCCGATCGCAGACAACAGTACCGGAGATGTCGCCGCTGACTCCTACCACAATGTCGACAGGGACGTGGAGATGATGCGGGAGTTAGGCCTCGACGCCTATCGCACCTCCCTGTCCTGGTCACGGATCCTGCCCACAGGATTCGCCGACCACATCAACGAGGCTGGAGTCAACTACTACAACCGACTTTTCGATGAGATGCTCAAGTATAACATCCAACCCGTGATAACCCTCTTCCACTGGGACTTACCGCAGCCCCTTCAAGACCTGGGCGGGTTGGCCAACCCCCTCTTTCCCGAGTGGTTCGAAGATTACTCCCGAGTTGCCTACGAGGCGTTCGGGGATAGAGTAAAGTTTTGGGTCACGTTCAACGAGCCAACCCAGATTTGCCTCCTCGGTTATGGCGACACTTCCATGGCACCAGAGCTAGGGGCTGGTGGAATTGGCGAGTACATGTGCGCTAAGAATCTGCTTCTGGCTCACGCTAAAGCCTATCATTTGTATGATAGTGAGTACAGGAGCACTCAAGCTGGGCAAGTGGGCTTGGCGATACATATCACCTACAATCAACCCTTGACTGATAGTGAGGAAGATAGAATTGCTTGTGAATTGTATAACCAGGCTACGATCGGTATTTACAGTGATCCCATCTTCGCCGCTGAGCCCGGCTGGCCAAAAGAGCTGAAACAAAAGATCGCTGAAAAAAGCGCAGCCCAAGGTTTCCCAAGGTCAAGGTTACCGGAACTGACAGATGAAGAGGCGATCTTCATCCATGGGTCCTCGGACTACTATGGAATTAACCACTACACCACTCTGAAGGTGGCTTCCGCAGTGAATGTGAGTGGGAGGAGAGTGCCATCGTTGATGGATGACATTGATGCTGAGGCTATCCGGAACGGCACTGGGGAAAATTGGATGCAAGCTGCTTCGTATTGGCTATTCCTTCATCCAGAAAGCATGTCCAAGGTATTTGAAATGTTAGAAGAGAGGTACAATGACCAAGTCTACTATATAATGGAGAGTGGCCGGTCGACTGCCGGCGGGTTGGTGGATGACGACCGAGTGACGTATTACAGGACTGTTCTGAACAGTATGTTGGATGCTATGGAGGAGGGGGCTCGCGTCAAGGGCTACATGGCTTGGAGCCTGATGGATAATTTTGAGTGGAAGCGGGGATACACAGAGAAGTTTGGTTTCTACGAAGTGGACCTGGAGGACCCAGCTCGCACCAGAACTCCGAGGAAGTCGGCATTTGTTTACAAACACATCGTAAAGACAAGAACCATTGATCCTGATTATGAGCCAGAAGAATTTGTCATGTCGATTCATGAATAGGGTTTCTGGTCGAGAATTCTCGAGCTCGAGAAGATTCGAGAAATTCGGCTTGGTTCGAGACGAGAAAAAAATCTGAAGACTCGAGAATTTCTCGAGCCTTGAGATAAAAATTAAGAATTTTCAATTCTTATTTTCCTGCGCCGCTTAACAACACACACGCATACCTACCAATC |
| > *Px008848* full-length amplification in AD strain  CCTATATCTCTCGTCTCACCAATAAACCATGACATTCAGCTTATCCTATCGTGTTATAGTCCTGGTTGCATTGCTAAGTGTAGCGTCGGGAAAAACAAAAGATGTGCACAGCAGAAAGTTTCCCGATGACTTTTTGTTTGGGGCAGCCACGGCGTCTTACCAGGTAGAAGGCGCTTGGAATGAGGATGGTAAAGGCGAAAACATTTGGGATCGTTTGACTCACAGAGACCCTTCGCCGATCGCAGACAACAGCACCGGAGATGTCGCCGCTGATTCCTACCACAATGTGGACCGGGACGTGGAGATGATGCGGGAGTTAGGCCTCGACGCCTATCGCACCTCCCTGTCCTGGTCACGGATCCTTCCCACAGGATTCGCCGACCACATCAACGAGGCTGGAGTCAACTACTACAACCGACTTTTCGATGAGATGCTCAAGTATAACATCCAACCCGTGATAACCCTCTTCCACTGGGACTTACCGCAGCCCCTTCAAGACCTGGGCGGGTTGGCCAACCCCCTCTTTCCCGAGTGGTTCGAAGATTACTCCCGAGTTGCCTACGAGGCGTTCGGGGATAGAGTAAAGTTTTGGGTTACATTCAATGAGCCATCCCAAATTTGCCGCCTCGGCTATGGAGACAAATCCATGGCACCAGAGCTAGGGGCTGGTGGTGTTGGCGAGTACATGTGCGCTAAGAATCTGCTTCTGGCTCACGCTAAAGCCTATCATTTGTATGATAGTGAGTACAGGAGCACTCAAGCTGGGCAATTGGGCTTGGCGATACATATCACCTATAATCAACCCTTGACTGATAGTGAGGAAGATAGAATTGCTTGTGAATTGTATAACCAGGCTACGATCGGTATTTACAGTGATCCCATCTTCGCCGCTGAGCCCGGCTGGCCAAAAGAGCTGAAACAAAAGATCGCTGAAAAAAGCGCAGCCCAAGGTTTCCCAAGGTCAAGGTTACCGGAACTGACAGATGAAGAGGCGATCTTCATCCATGGGTCCTCGGACTACTATGGAATTAACCACTACACCACTCTGAAGGTGGCTTCCGCAGTGAATGTGAGTGGGA |
| >*Px008849* full-length amplification in FZ strain  CCTGTAATAAAGAAGGCTAATCTTAATGTGAATTTACTAAAGTTAACTAAATTGTTCAGATTAAAGAAGAAGTTGTGACTATGAAGACTGTGATTTTTGTGATCTGCGTGGTGTCAAGCTCCCCGGGCCTGGCGAGGGTCGGCCCTGCCGGGAGACAGTTCCCGCCAGACTTCCTGTTCGGGACCTCTTCAGCATCATACCAGGTCGAGGGTGCTTGGAACGAGGATGGCAAGGGCGAGAGTATATGGGACAGATTCGTCCACCGAGACCCACCACCAGCTAAAGATGGCAGCACCGGCGACGTGGCTAACGACTCTTACCACAAGTACAAACGGGACATACAGATGTTAAGAGAACTGGGGGTCAATACCTACCGCTTCTCCATATCCTGGACTCGCATCCTTCCAACTGGCTTCTCCAACTATATAAACCCCCTTGGAGTCCAATACTATAACAATGTTATAGATGAGCTGCTCAAGTATAATATAGGGCCGATAGTGACGATATTCCACTTCGATCTGCCACAGTCTTTACAAGATCTAGGTGGCTTTGCCAATCCTCTGATAGAAGGCTGGTTTGAGGATTACGCTAGAGTTGTCTTTGGATTATACGGGGATAGAGTGAAGAAGTGGATCACGATCAATGAACCGAGGGAAACCTGCAGTGAGGCTTACGGCACTGTGACGTCGGCACCTGGCCTGAATTTCTCCGGGTTTGCAGATTATCTCTGCGCGAAGTATGTGCTGATATGCCATGCCAGTGCCTATCGCCTGTATGATAGAGAGTTCAGGGCGTCACAAGGAGGGGAGGTTGGGATAGCATACAGTGCCAGCTGGTACGCGCCAGCGACTGATTCTGTTGAGGATGAGTTAGCTACAGAGTTGAAACGACAATCGGAGTTAACAATATACGTAGACCCGGTATTCTCCGAAGAGGGGGGGTTCCCAGCAGAGTTGTCAACCAGAATAGCACAGAAGAGCGCTGAGCAGGGATACCCATTCTCACGATTACCAGCTTTCACAGACGAGGAGAAGGCATTCGTGAGAGGAACAGCGGATTTCCTTGGCGTCAACCATTACTGCTCCTTCCTTATATCAGCTACTAAGAACCTGCAGGAAAACCCCCGTGTGCCGTCGTTGGCTGACGATGTGAATGTGGGTCTGGTGATCCCGGACGAGTGGCCACACTCGGCTTTGAGCTTCATGGCGAGATCCCCCAACAGCCTATTCAACGTGCTATCATATTTTAACGCCAGATACAACAAAAACATAACGTATTACATAACGGAGAACGGTTGGGCTGTGGACGACGGTCTAGAAGGCGATAGGATCGCCAACTACAGAGGCAACCTAGAAGGAGTTCTTGACAGTTTGGACGCTGGCATCAGAGTGAAGGGGTTCATGGCATGGACTCTCATGGATAATTATGAGTGGATCAGTGGTTTTAGTGTTAAGTTTGGTCTCTATCGAGTGGACAGAAGTGATAGTGAGCTTCGCAGAATACCGCGGGAAACAGCCTTTGTTTATAAGGAAATTATAAAAACTCGACAGATTGATCACAAGTATTACCCTACTACGAAAGAAATGACGATAGATGACGGACATTAGTATTACCTATGTAGTTAAGTAGGTACTTAATATGATTTTATACCAAAATTAATTTAAATAGCTAACTACTACCTAGTTAGGTAGTTAGTTAGTTATACTTTCACGAAAACAAGCAGCGGGCTTTTCTTTCCTT |
| > *Px008849* full-length amplification in AD strain  TCGAGTTTTAGCAGATCCTGTAATAAAGAAGGCTAATCTTAATGTGAATTTACTAAAGTTAACTAAATTGTTCAGATTAAAGAAGAAGTTGTGACTATGAAGACTGTGATTTTTGTGATCTGCGTGGTGTCAAGCTGCCCGGGCCTGGCGAGGGTCGGCCCTGCCGGGAGACAGTTCCCGCCAGACTTCCTGTTCGGGACCTCTTCAGCATCATACCAGGTCGAGGGTGCTTGGAACGAGGATGGCAAAGGCGAGAGTATATGGGACAGATTCGTCCACCGAGACCCACCACCAGCTAAAGATGGCAGCACCGGCGACGTGGCTAACGACTCTTACCACAAGTACAAACGGGACATACAGATGTTAAGAGAACTGGGGGTCAATACCTACCGCTTCTCCATATCCTGGACTCGCATCCTTCCAACTGGCTTCTCCAACTATATAAACCCCCTTGGAGTCCAATACTATAACAATGTTATAGATGAGCTGCTCAAGTATAATATAGAGCCGATAGTGACGATATTCCACTTCGATCTGCCACAGTCTTTACAAGATCTAGGTGGCTTTGCCAATCCTCTGATAGAAGGCTGGTTTGAGGATTACGCTAGAGTTGTCTTTGGATTATACGGGGATAGAGTGAAGAAGTGGATCACGATAAATGAACCGAGGGAAACCTGCAGTGAGGCTTACGGCACTGTGACGTCGGCACCTGGCCTGAATTTCTCCGGGTTTGCAGATTATCTCTGTGCGAAGTATGTGCTGATATGCCATGCCAGTGCCTATCGCCTGTATGATAGAGAGTTCAGGGCGTCCCAAGGAGGGGAGGTTGGCATAGCTTACAGTGCCAGCTGGTATGCGCCTGCTACTGATTCTGTTGAAGATGAGCTCGCTACGGAGTTGAAACGACAATCGGAGTTAACAATATACGTAGACCCGGTATTCTCCGAAGAGGGGGGTTTCCCAGCAGAGCTGTCAACCAGAATAGCACAGAAGAGCGCTGAGCAGGGCTACCCATTCTCACGATTGCCAGCTTTCACTGAAGAGGAGAAGGAATTTGTCAGAGGAACAGCCGATTTCCTTGGTGTCAACCATTACTGCTCCTTTCTCATATCAGCTACTAAGAACCTACAGGAGAACCCCCGTGTACCGTCGTTGGCTGACGATGTCAATGTGGGTCTGGTGATCCCGGACGAATGGCCACACTCGGCTTTGAGCTTCATGGCGAGATCCCCCAACAGCCTATTCAACGTGCTATCATATTTTAACGCCAGATACAACAAAAACATAACATATTACATAACTGAGAACGGCTGGGCGGTTGACGAGGGTCTAGAAGGCGATAGGATCGCCAACTACAGAGACAATCTAGAAGGAGTTCTTGACAGCCTGGACGCTGGCATCAGAGTGAAGGGGTTCATGGCATGGACTCTGATGGATAATTATGAGTGGATCAGTGGTTTTAGTGTAAAGTTTGGTCTCTATCGAGTGGACAGAAGTGATAGTGAGCTTCGCAGAATACCGCGTGAAACTGCCTTTGTTTATAAGGAAATTATAAAAACTCGACAGATTGATCACAACTATTACCCCACTATGAAAGAAATGACGATAGATGACGGACATTAGTACCTACCTAGTTAAGTAGGTACTTAATATGATTTTATACCAAAATTAATTAAAATACCTAGTTACTACCTAGGTAGTTAGTTATACTTTCACGAAAACAAGCAGCGGGCTTTTCTTTCCTTTTGATATCTT |
| >*Px006054* full-length amplification in FZ strain  TCGCCAGTTCGTTGTTGATAGCCAACAGCGGAGGACGACCATGGCGGCTAAATGGACGATCATCGCAGCCTTAGCCCTTTGCCACACGGCATTGGTTGAGTACACCAAATTCCCTGAAGGTTTCACTTTCGGAGTCGCCACCGCTGCTCATCAAATTGAGGGAGGCTGGAACGAAAATGGTAAAGGTGAGAACGTGTGGGACCACCTCAGCCACAACCGTCCTGAGCTGATAGCTGATGGTTCAAGCGGAGACGTAGCCACTGACTCGTACCACAGATACCGTGAAGATGTGGAGGAGCTGGCGTACCTCGGGGTGGACTTCTACCGCATGTCGTTCTCGTGGGCGCGCCTGCTGCCGAATGGACGCATTGATAATGTGAACGCTGACGGCGTCCGCTATTACAACGAGCTGCTTGATGCTTTGGCTGCGCATAATATCGAGCCTTTGGTGACCCTCTTCCACTGGGACTTGCCGCAAGTGCTGCAAGACCTGGGCGGCTGGGCCAACCCGCACATGATCGACTACTTCCGCGACTACGCTGACTTCTGCTACAAGACCTTCGGAGGCAAGATCAAGTCCTGGATCACCTTCAACGAGCCCTACGAAATCTGCGAGGACGCTTACGGAGACATCCTCAAAGCACCAGCAGTTGACAGCCACGGTGTCGGGAACTACCTCTGCAGTGATACGTTGCTGAAGGCCCATGCTGAGGCCTATCACCTGTACAATGAGACGTACAAGCCGGTGCAAGATGGCAAGGTCATGATCTCGATCAACTCCATCTGGTACGAGCCTAAAGACCCCAGCAACGCTGAGCAGGTGGTGTTGGCTGAAACTGCTAATCAGTTCAAGTTCGGTTGGTTCGCGCACCCCATCTTCTCGAAGGAGGGCGGCTACCCCGCCGTCATGATCGAGAACGTCGCCCGCAACAGCGAGGCCGAAGGTCTGAAGCGCTCCCGGCTGACCCAGTTCGACGAGTACTGGACGGCCCGCATCAAGGGCACCTCGGACTTCCTCGGCATCAACCACTACACCACGCACCTGGTGACGGGCGCGGGCGTCGACCCCGGGGCGAAGTCGCCGTCCTGGCTGAAGGATGTGGGGGCAGTTGTGTCTACTGATGTCGGCACGGATTCCGCGTCCGCTTGGCTTAGGGTGGTCCCCTCCGGGTTCGCGAACCTGCTCCGCTGGTGCAAGCGCTCCTACTCCGACCCTCCGATCTACATCACGGAGAACGGCTACTCCGACCGCGGGGAGCTGATGGACTACAAGCGGATCAGCTACTTCAATGACTACCTGTCGGAGATCCTGAACGTGATCAACAACGACGGCGTGCGCGTGCTGGGCTACACGGCCTGGACGCTCATGGACAACTTCGAGTGGCGAGCCGGGTTCTCGGAGCGTTTCGGTTTATACCACGTGAACATCACGGACCCGGCGCTGCCTCGCACTGCGAAGCTCTCAGCCGACTACTTCAAGCAACTCATCGCCAACAGAGAGCTTCCCAAAGACGACAAATACAAAGAGCCAGCCAGACACTGAAGCTCGCACCACTAACA |
| >*Px006054* full-length amplification in AD strain  TCGAGTTTTTAGCAGATTCGCCAGTTCGTTGTTGATAGCCAGCAGCGGAGGACGACCATGGCGGCTAAATGGACGATCATCGCAGCCTTAGCCCTTTGCCACACGGCATTGGCTGAGTACACCAAATTCCCTGAAGGTTTCACTTTCGGAGTCGCCACCGCTGCTCATCAAATTGAGGGAGGCTGGAACGAAAATGGTAAAGGTGAGAACGTCTGGGACCACCTCAGCCACAACCGTCCTGAGCTGATAGCTGACGGTTCAAGCGGCGATGTGGCCACAGACTCGTACCACAGATACCGTGAAGATGTGGAGGAGCTGGCGTACCTCGGGGTGGACTTCTACCGCATGTCGTTCTCGTGGGCGCGACTGCTGCCGAATGGACGCATTGATAACGTGAACGCTGACGGCGTGCGCTATTACAACGAGCTTCTTGATGCTTTGGCTGCGCATAATATTGAGCCTTTGGTGACCCTCTTCCACTGGGACTTGCCGCAAGCGCTACAAGACCTGGGCGGCTGGGTCAACCCGCACATGATCGACTACTTCCGCGACTACGCAGACTTCTGCTACAAGACCTTCGGAGGCAAGATCAAGTCCTGGATCACCTTCAACGAGCCCTACGAAATCTGCGAGGACGCTTACGGAGACATCCTCAAAGCACCAGCAGTTAACAGCCACGGTGTTGGAAACTACCTCTGCAGTGATACGCTGTTGAAGGCTCATGCTGAGGCCTATCATCTGTACAATGAGACGTACAAGTCGGTGCAAGATGGCAAGGTCATGATCTCGATCAACTCCATCTGGTACGAGCCTAAAGATCCCAGCAACGCTGAGCAGGTGGTGTTGGCTGAAACTGCTAACCAGTTCAAGTTCGGTTGGTTCGCACATCCCATCTTCTCGAAGGAGGGCGGCTACCCCGCCGTCATGATCGAGAACGTAGCTCGCAACAGCGCGGCTGAAGGTCTGAAGCGTTCCCGGCTGACCCAATTCGACGAGTACTGGACGGCGCGCATCAAGGGCACCTCTGACTTCCTCGGCATCAACCACTACACCACGCACCTGGTGACGGGCGCGGGCGTCGACCCCGGGGCGAAGTCGCCGTCCTGGCTGAAGGATGTGGGGGCAGTTGTGTCCACTGATGTGGGCACGGACTCCGCGTCCGCTTGGCTTAGGGTGGTCCCCTCCGGGTTCGCGAACCTGCTCCGCTGGTGCAAGCGCTCCTACTCCGACCCTCCGATCTACATCACGGAGAACGGCTACTCCGACCGCGGCGAGCTGATGGACTACAAGCGGATCAGCTACTTCAATGACTACCTGTCGGAGATCCTGAACGTGATCAACAACGAACTGGCGAATCTTTCTAGAAGATCTCCTACAATATTCTCAGCTGCCATGGAAAATCGATGTTCTTCTTTTATTCTCTCAAGATTTTCAGGCTGTATATTAAAACTTATATTAAGAACTATGCTAACCACCTCATCAGAACCGTTGTAGGTGGCGTGGGTTTTCTTGGCAATCGACTCTCATGAAAACTACGAGCTAAATATTCAATATGTTCCTCTTGACCAACTTTATTCTGCATTTTTTTTGAACGAGGTTTAGAGCAAGCTTCAGAACTGAGACAGGATTTTATTAAAAATTTAATTTTGAGAAGTTCAGATATAGCATCCATTTTTGCTT |
| >*Px006941* partial-length amplification in AD strain  CATGTGTGTCAAGAATGTGCTGATGGCCCATGGGAAGGCGTATAGGGTTTATGAGAGGGAGTTTAAGGGAAAATATTCGGGAAAACTATCGATGGCAAATCATTTCATGTGGTATGAAGCTGAGACGGAGAAGGATTTGGAAGTAGCCGAACAAATTAGGCAGTTTCAGTACGGCCTGTACTGCCACCCCATCTACTCGCAGGCGGGCGGCTGGCCGGCGGCGGTGGAGCGGCAGATCGCCGACAACAGCCGCAGAGAGGGTTACAGCTTCTCCCGGCTGCCGGGGTTCACGCAGGAGGAGAAGGACTTGGTCAGAGGTACCTTCGACTACATGGCAGTGAACCACTACAGCAGCAGGACGGTCCGCAAGCAGCAGTCAGCGGAACACATCCCAGCTTCCAACCAGTTCACTGCGAATGCAGAGTTTAACGCCACCTATGAGGGCAAGGAGGACTGGCCTAAGGGAAACTCCTTTTGGTTCTTGATAAACCCGGCCGGCCTCCGCAACCTGCTGGTGTGGCTGCGGAACGAGTATGGAGACATCGAGTATCTCATCACAGAGAACGGCTACTCTGACGTCACCACCAAGATCAATGATGACGATAGGGTTGACTATCATAGGGAGTATTTGAAACAGGTTCTTTTGGCCATAAAGCAAG |
| >*Px009428* same partial-length amplification in FZ and AD strains  GTTTATGAGAGGGAGTTTAAGGGAAAATATTCTGGAAAACTATCGATGGCAAATCATTTCATGTGGTATGAAGCTGAAACGGAGAAGGATTTGGAAGTAGCCGAACTAATAAGGCAGTTTCAGTACGGGCTGTACTGCCACCCCATCTACTCCCAGGAGGGCGGATGGCCGGCGGCGGTGGAGCGGCAAATAGCTGACAACAGCCGCAGAGAGGGCTACAGCTTCTCCCGGCTGCCGGGGTTCACGCAGGAAGAGAAGGAATTGGTCAGAGGTACCTTCGACTACATGGCAGTGAACCACTACAGCAGCAGGACAGTCCGGAAGCAGCCGTCAGCGGAACACATCCCTGCTTCCAACCAGTTCACGGC |
| >*Px009427* partial-length amplification in AD strain  CGTTCCCCCCCGGGTTCAGGTGGGGCGCCGCCACCGCCGCCTACCAGGTCGAGGGCGCATGGAACCAGAGTGACAAATCACCAAGCATATGGGACAAGGTGGTCCATGAGTACAACCACCTGATCCAGGACCGAAGCAATGGCGACGTGGCCTGCGACTCCTACCACCTCTGGAAGAGAGACATACAGATGGCCAAGGAATTGGGCCTTGACTTTTACAGATTCTCCATAGCCTGGACTCGCCTGATACCAAATGGCTTCTCGAACGCAATCAGCGAAGATGGCAAGAATTATTACAACAACCTCATCGACGGCCTCTTGGCTCACGGCATCCAACCGATGCCCACGTTGTACCACTGGGACTTACCTCAGAGGTTGCAAGATTTAGGTGGCTGGAGCAACCCGCTGGTAGCCGACTGGTTTGCGGACTACGCTCGCGTGGCCTTCTCCCTCTTCGGAGACCGCGTGCAGTACTGGATCACCATCAACGAGCCCACCATGGTCTGCGAACTGGGCTATGGCCTCGGCCTTGTGGCCCCCTTGATCAAGGA |
| >*Px000291* partial-length amplification in FZ strain  GATGACGTGCGCATCATACGGGATCTGGGCGTGTCCACGTATAGGCTGTCCATCTCTTGGAGCAGGATACTTCCAAACGGCACTGACAACTACATAAACGCCGAAGGAGTCCAATACTACCGCAACCTATTCGACGAGCTCGCAAAATACAACATCACACCAATGGTCACACTATTCCACTGGGACTTGCCCCAAATGTTCATGGACCTTGGCGGATGGACCAATCCCGAGATGGTGGATTATTTTGAGGACTACGCGAGAGTCGCGTTTAATCTGTTCGGGGATGTCGTGAAGATTTGGACGACCATTAATGAGCCGCATCAGCATTGTTATAATGGCTACGGCACAGATTACTTCGCACCCGCACTAGAATCATACGGGGTGGGCGAATACCTGTGCGACCACTACATACTTCTAGCACATGCTAGAGTGTACCATCTGTATGATAGGGAATTCCGGCCGAAATACAAAGGAAAAATCGGGATAACCCTGGACGCTTTCTGGGCCGATCCTCTCGACCCCACGAAGGAAGAAGACCGAGAGGCAGCAGAAAGATATATGATGATGAATCTAGCCCGCTACGCGCACCCCATCTACTCGGCGGAGGGCGACTACCCGGCCGTGGTGCGCGCGCGCATCAACACCATCAGCCAGCTGCAGGGCTTCCCGCGCTCGCGCCTGCCGTACTTCACGGCTCAGGAGATAGAGGACCTCCGTGGCTCCTCCGACTTCTTCGGACTGAACCACTACACCACGTTCCTGATGACTCCGTCCCGCATGAAAAAGGGG |
