# Supplementary material for: An inducible gene from glycoside hydrolase one family of Plutella xylostella decreases larval survival when feeding on host plant
Source: Front Physiol. 2022 Oct 20;13:1013092. doi: 10.3389/fphys.2022.1013092 (PMC9632345; doi:10.3389/fphys.2022.1013092)
Supplement: Supplementary file 6 [file DataSheet1.PDF]

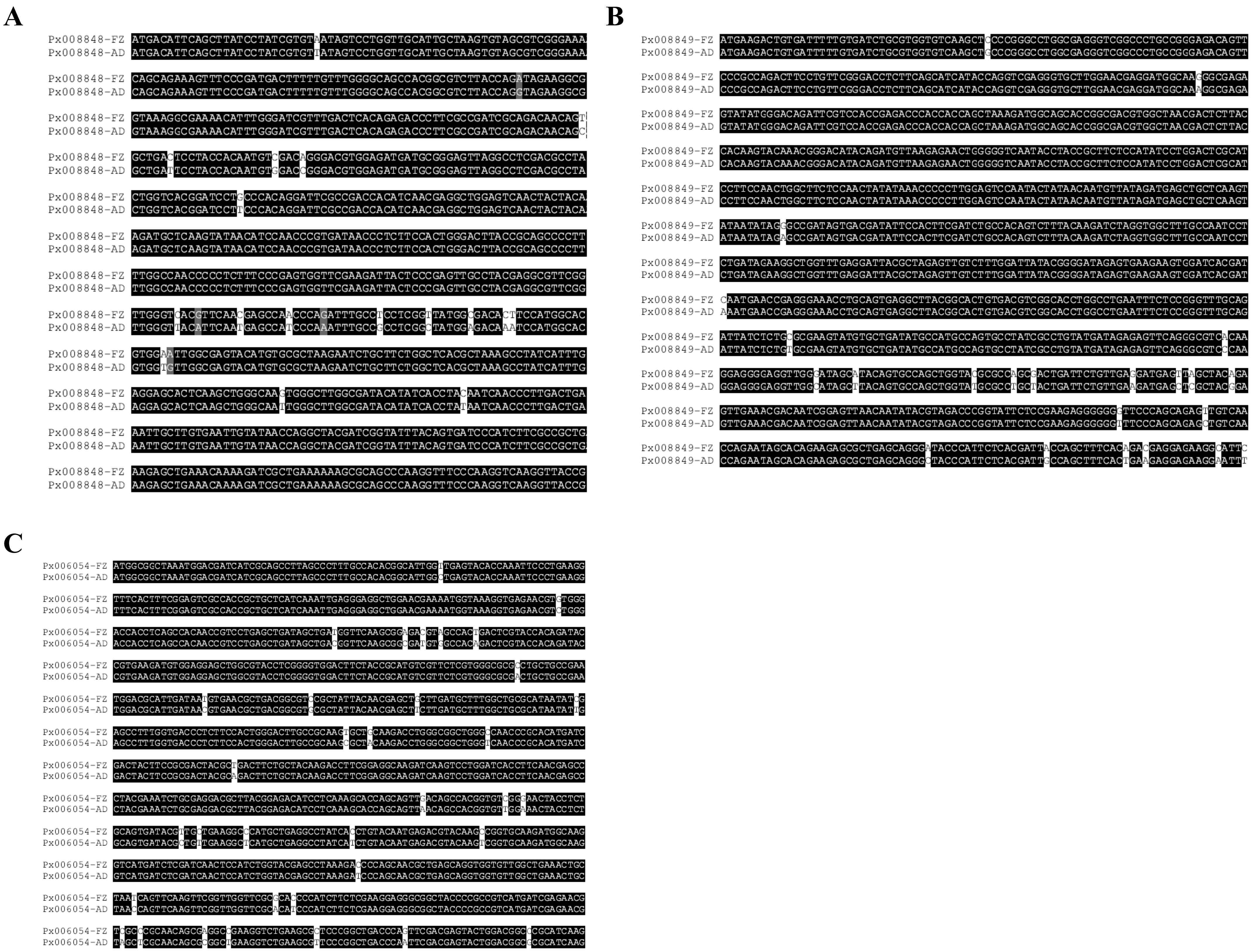
A
Px008848-FZ
Px008848-AD
B
Px008849-FZ
Px008849-AD
C
Px006054-FZ
Px006054-AD
