# Supplementary material for: An inducible gene from glycoside hydrolase one family of Plutella xylostella decreases larval survival when feeding on host plant
Source: Front Physiol. 2022 Oct 20;13:1013092. doi: 10.3389/fphys.2022.1013092 (PMC9632345; doi:10.3389/fphys.2022.1013092)
Supplement: Supplementary file 7 [file DataSheet5.PDF]

Deduced amino acid sequence of Px008848

1	MKTVIFVICV	VSSSPGLARV	GPAGRQFPPD	FLFGTSSASY
41	QVEGAWNEDG	KGESIWDRFV	HRDPPPAKDG	STGDVANDSY
81	HKYKRDIQML	RELGVNTYRF	SISWTRILPT	GFSNYINPLG
121	VQYYNNVIDE	LLKYNIGPIV	TIFHFDLPQS	LQDLGGFANP
161	LIEGWFEDYA	RVVFGLYGDR	VKKWITINEP	RETCSEAYGT
201	VTSAPGLNFS	GFADYLCAKY	VLICHASAYR	LYDREFRASQ
241	GGEVGIAYSA	SWYAPATDSV	EDELATELKR	QSELTIYVDP
281	VFSEEGGFPA	ELSTRIAQKS	AEQGYPFSRL	PAFTDEEKAF
321	VRGTADFLGV	NHYCSFLISA	TKNLQENPRV	PSLADDVNVG
361	LVIPDEWPHS	ALSFMARSPN	SLFNVLSYFN	ARYNKNITYY
401	ITENGWAVDD	GLEGDRIANY	RGNLEGVLDS	LDAGIRVKGF
441	MAWTLMDNYE	WISGFSVKFG	LYRVDRSDSE	LRRIPRETAF
481	VYKEIIKTRQ	IDHKYYPTTK	EMTIDDGH	
